# Novel transcriptome assembly and comparative toxicity pathway analysis in mahi-mahi (*Coryphaena hippurus*) embryos and larvae exposed to Deepwater Horizon oil

**DOI:** 10.1038/srep44546

**Published:** 2017-03-15

**Authors:** Elvis Genbo Xu, Edward M. Mager, Martin Grosell, E. Starr Hazard, Gary Hardiman, Daniel Schlenk

**Affiliations:** 1 Department of Environmental Sciences, University of California, Riverside, CA 92521, USA; 2 Department of Biological Sciences, University of North Texas, Denton, TX 76203, USA; 3 Department of Marine Biology and Ecology, University of Miami, Miami, FL 33149, USA; 4 Center for Genomic Medicine, Medical University of South Carolina, Charleston, SC 29403, USA; 5 Computational Biology Resource Center, Medical University of South Carolina, Charleston, SC 29403, USA; 6 Departments of Medicine & Public Health Sciences, Medical University of South Carolina, Charleston, SC 29403, USA; 7 Laboratory for Marine Systems Biology, Hollings Marine Laboratory, Charleston, SC 29412, USA.

## Abstract

The impacts of Deepwater Horizon (DWH) oil on morphology and function during embryonic development have been documented for a number of fish species, including the economically and ecologically important pelagic species, mahi-mahi (*Coryphaena hippurus*). However, further investigations on molecular events and pathways responsible for developmental toxicity have been largely restricted due to the limited molecular data available for this species. We sought to establish the *de novo* transcriptomic database from the embryos and larvae of mahi-mahi exposed to water accommodated fractions (HEWAFs) of two DWH oil types (weathered and source oil), in an effort to advance our understanding of the molecular aspects involved during specific toxicity responses. By high throughput sequencing (HTS), we obtained the first *de novo* transcriptome of mahi-mahi, with 60,842 assembled transcripts and 30,518 BLAST hits. Among them, 2,345 genes were significantly regulated in 96hpf larvae after exposure to weathered oil. With comparative analysis to a reference-transcriptome-guided approach on gene ontology and tox-pathways, we confirmed the novel approach effective for exploring tox-pathways in non-model species, and also identified a list of co-expressed genes as potential biomarkers which will provide information for the construction of an Adverse Outcome Pathway which could be useful in Ecological Risk Assessments.

Crude oil spills in critical fish spawning and nursing habitats are a recurrent worldwide problem^1^,^2^,^3^. The Deepwater Horizon (DWH) incident in 2010, the largest marine oil spill in U.S. history, resulted in exposure of many spawning pelagic fish species of economical and ecological importance, such as mahi-mahi (*Coryphaena hippurus*), bluefin (*Thunnus thynnus*) and yellowfin tuna (*Thunnus albacares*)^4^,^5^,^6^. The DHW oil is a complex chemical mixture, with polycyclic aromatic hydrocarbons (PAHs) and their alkylated homologues being the main toxic components to fish embryos^7^,^8^. The composition and structure of individual PAHs in the water column can be significantly altered by weathering processes, and it has been shown that weathered surface slick oil (3- and 4-ring PAHs enriched) is more toxic than non-weathered source oil to mahi-mahi on a ∑PAH basis at different levels from molecular to physiological functions^9^,^10^. Numerous studies have investigated the developmental toxicity of crude oil to fish embryos and larvae and identified a variety of abnormalities such as cardiac function, kidney development, formation of the craniofacial skeleton (eye and jaw), nervous system as well as reduced swimming performance^7^,^10^,^11^,^12^,^13^. These impacted functions and biological processes may contribute to delayed mortality and declined population as previously observed in pink salmon (*Oncorhynchus gorbuscha*) exposed to crude oil^14^,^15^. Consequently, a baseline understanding of crude oil toxicity to the fish species native to the oiled pelagic zone is essential for ecological risk assessment, regulatory decision making, and spill response planning.

Despite the relatively rich literature on functional and morphological impacts of crude oil on fish embryonic development, the molecular initiating events preceding these effects are less understood. A recent study of Atlantic haddock (*Melanogrammus aeglefinus*) embryos exposed to crude oil found a downregulation of mRNAs encoding cardiac rapid potassium channel, as well as the sodium/calcium exchanger (*ncx1*) that regulates intracellular calcium levels during E-C coupling^16^. Another study in mahi-mahi focused on the developmental cardiotoxicity of crude oil exposure targeted ten molecular indicators of cardiac stress and injury, such as atrial and ventricular myosin heavy chains (*amhc*/*myh6* and *vhmc*/*myh7*), cardiac myosin light chain 2 (*cmlc2*/*myl7*), B-type natriuretic peptide (*nppb*), as well as transcription factors *nkx2.5* and *tbx5*^17^. Our previous study by RNA sequencing and physiological assessment demonstrated that slick oil exposure resulted in pronounced perturbations in metabolism, steroid biosynthesis, vision, and AhR genes suggesting other targets in addition to the heart may be involved in the developmental toxicity of DHW oil^10^. Given the increasing evidence of morphological and physiological impacts of crude oil on a number of fish species, a comprehensive and accessible genomic database is urgently needed for target species in oiled regions to advance our understanding of the toxic molecular mechanisms of crude oil. Here, we focus on mahi-mahi, *Coryphaena hippurus*, a resident species in the DWH-oiled water, as a target for investigating the transcriptomic responses reflective of developmental toxicity of crude oil. This species is a highly commercially important fish widespread in tropical and temperate waters and occurs in the Atlantic, Indian, Pacific Oceans and the Mediterranean^18^, which thus makes an excellent candidate for understanding the impact of the recurrent oil spills worldwide.

The present study aimed to establish, for the first time, a *de novo* global transcriptome database from embryos and larvae of mahi-mahi exposed to water accommodated fractions (HEWAFs) of two oil types (slick/weathered oil and non-weathered source oil) using high throughput sequencing (HTS) and *de novo* assembly technology. In contrast, our previous study used a commercially available reference-transcriptome-guided automated OnRamp approach that failed to provide sequence information for *de novo* transcriptome assembly efforts for mahi-mahi^10^. To confirm prior molecular signatures and provide an accessible transcriptome data base, differential expression analysis was conducted in 96 hpf larvae exposed to slick oil by the *de novo* approach, and downstream gene ontology (GO) terms were compared to the 24, 48 and 96 hpf mahi-mahi exposed to both source and slick oil as determined by the earlier OnRamp approach. Since a number of individual genes were not commonly regulated among different developmental stages (24, 48, 96 h), oil types (slick and source oil) and transcriptome analytical approaches (*de novo* and OnRamp), we also considered state-of-the-art comparative toxicity pathway (tox-pathway) analysis, which closely related to the concept of adverse outcome pathways (AOP). Furthermore, the common tox-pathways and a list of common responsive genes were identified as potential biomarkers to predict crude oil toxicity or recovery in mahi-mahi.

## Results

### *De novo* assembly of mahi-mahi transcriptome and annotation

**Figure 1:**
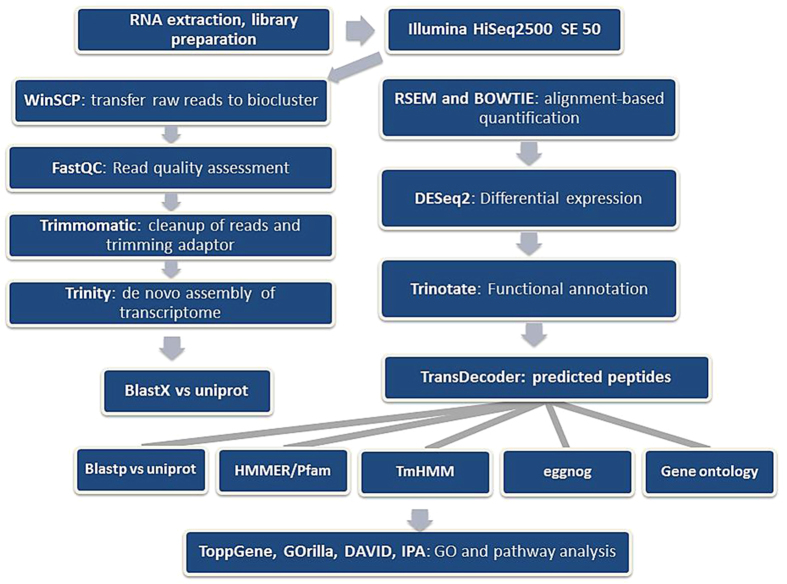
Schematic flow diagram illustrating the workflow employed for sequencing, *de novo* assembling and annotating the mahi-mahi transcriptome and for determining changes in gene expression, GO terms and regulated pathways in DHW oil exposed mahi-mahi embryos and larvae.

Schematic flow diagram illustrating the workflow employed for sequencing, *de novo* assembling and annotating the mahi-mahi transcriptome is presented in Fig. 1. A total of 1,647,902,734 Illumina HiSeq reads from mahi-mahi embryos and larvae were generated. After trimming the adapters, 42,447,657 bases were assembled with Trinity resulting in 60,842 transcript contigs with an average length of 711 bp and an N50 of 1,093 bases, 50% of which were annotated to known protein/nucleotide sequences by BLAST (e-value < 1 × 10^−5^) (Table 1). 17,370 HMMER/PFAM protein domains (Pfam), 3,168 predicted transmembrane regions (TmHMM), 26,444 non-supervised orthologous groups of genes (eggNOG), and 29,007 GO_blast were determined using Trinotate pipeline (Table 1). The final transcriptome assembly provided a high quality template for global gene expression profiling in this study and also provided a valuable resource for wider and deeper molecular research on mahi-mahi, which is an ecologically and economically important fish species with no existing genomic data.

### Quality of Gene Differential Expression Data

The unexposed control samples clustered separately from the slick oil treated sample, indicating global transcriptomic differences between the two sets (Fig. S1).

### Differentially expressed genes (DEGs)

**Figure 2:**
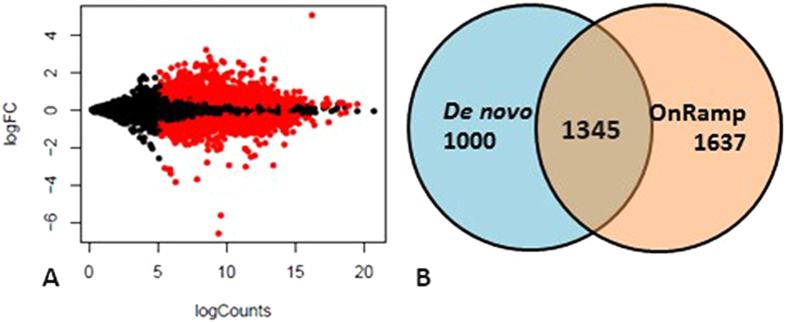
Plot of normalized mean counts (expression) versus log2 fold change for untreated versus treated comparison (**A**). The X-axis plots normalized logCounts and the Y-axis is the log2 fold change (FC). Black dots represent non-significant genes, whereas red dots indicate significant differentially expressed genes (q < 0.05). (**B**) Venn diagram displaying the number of differentially expressed genes in 96 hpf larvae after slick oil exposure and the overlay between these gene lists identified by *de novo* and OnRamp approaches.

In 96 hpf mahi-mahi larvae exposed to slick oil, a total of 2,345 genes were significantly differentially expressed at a False Discovery Rate (FDR) < 0.05 (Fig. 2A), (43%) of which overlapped with the differentially expressed genes previously identified by the Onramp approach (Fig. 2B). Transcripts of the cytochrome P450 *cyp1a1*, a phase-I enzyme regulated by the Aryl hydrocarbon receptor pathway (AhR), catalyzes the oxidative metabolism of endogenous and exogenous substrates such as PAHs, and was consistently the most strongly upregulated gene (30 fold by *de novo* approach and 26.5 fold by OnRamp approach). Methylsterol monooxygenase 1 (*msmo1*), functions in cholesterol biosynthesis, exemplified another top upregulated gene (4.4 fold by both *de novo* and OnRamp approaches). The expression patterns of nine selected genes in 96 hpf larvae after slick oil exposure were verified by RT-qPCR (quantitative real-time PCR)^10^.

### Gene Ontology (GO) terms analysis

*de novo* and OnRamp were consistent identifying significantly enriched GO categories (molecular function, biological process, pathway and phenotype) uncovered by both ToppGene and GOrilla (Tables S1 and S2). Both approaches revealed RNA binding and ATP binding as the predominant transcripts altered by slick oil exposure (>400 genes from input), which were also the most significantly enriched terms by DAVID against the zebrafish reference database (Table S3). The top overlapping biological processes and pathways impacted by slick oil exposure included transcripts involved in carboxylic acid metabolic process, cellular amino acid metabolic process, oxoacid metabolic process, ribosome biogenesis, cell development, metabolism, metabolism of amino acids, DNA replication, cholesterol biosynthesis, and synthesis of DNA. The significantly enriched common phenotypes included embryonic lethality, abnormal muscle morphology, abnormal muscle physiology, abnormal embryo development, abnormal cardiovascular system morphology, and decreased body size. Other biological processes and molecular functions that were more strongly enriched by *de novo* than by OnRamp included hydrolase activity, cellular lipid metabolic process, transmembrane signaling receptor activity, mitotic cell cycle process. Similar to ToppGene and GOrilla that are based on human/mouse references, DAVID topped ribonucleoprotein complex biogenesis, ribosome biogenesis, mitotic cell cycle, steroid metabolic process and heart development using zebrafish genome as background (Table S3). The top 20 GO terms by *de novo* approach are shown in Fig. S2.

### Ingenuity pathway analyses (IPA)

**Figure 3:**
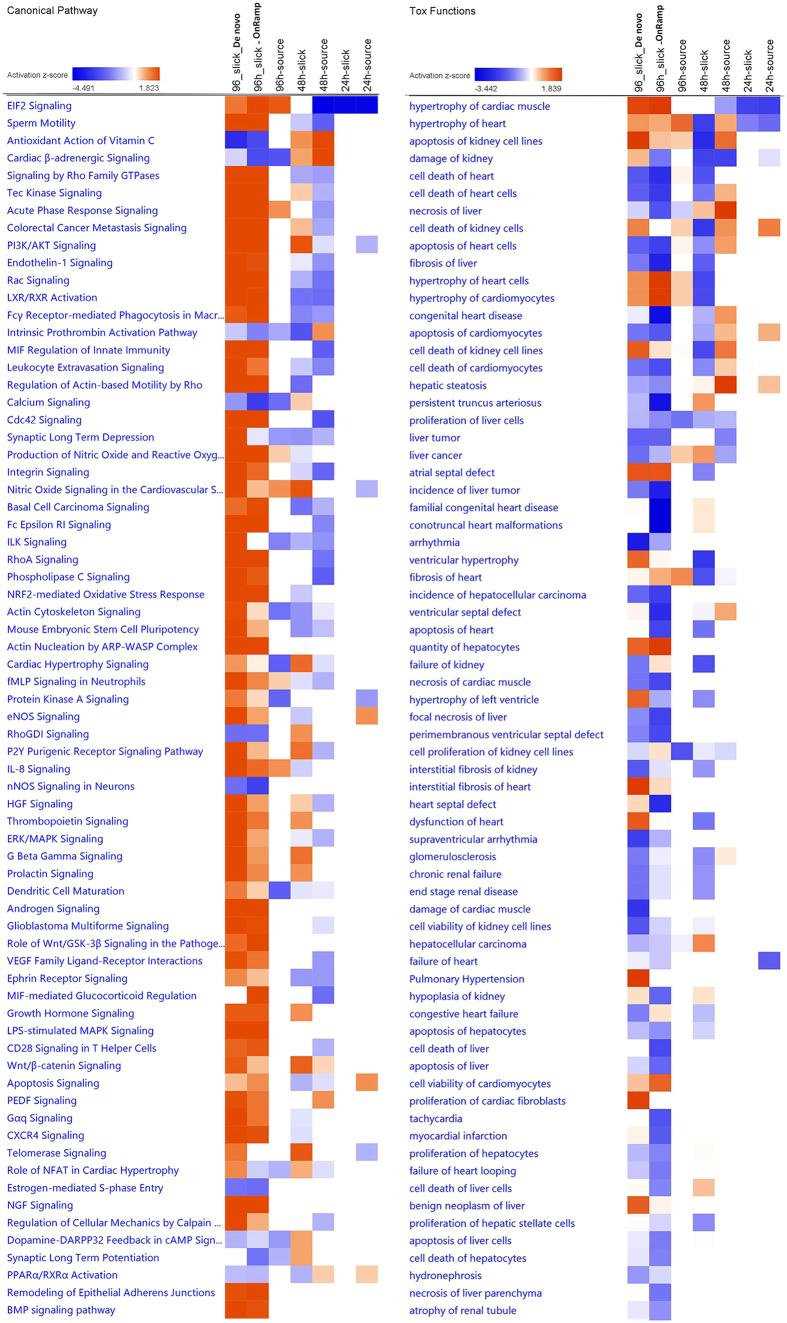
Heat maps clustering *de novo* (96h_slick) and OnRamp approaches (96h_slick, 96h_source, 48h_slick, 48h_source, 24h_slick, 24h_source) in canonical pathways and tox functions. Orange indicates the z-score is positive. IPA predicts that the biological process or function is trending towards an increase when Z-scores ≥ 1.3. Blue indicates z-score is negative. IPA predicts that the biological process or function is trending towards a decrease when Z-scores ≤ −1.3.

Canonical pathways and toxicity functions in mahi larvae at 96 h after slick oil exposure were analyzed by the *de novo* approach, and compared to responses 24, 48 and 96 h after both slick and source oil exposure by OnRamp. As expected, the 96 h_slick_*De novo* and the 96h_slick_OnRamp showed the highest similarity of impacted canonical pathways as well as toxicity functions (Fig. 3), reflecting similar pathway profiles with the two different approaches of analyses. The representative commonly affected canonical pathways and functions included EIF2 signaling, cardiac β-adrenergic signaling, signaling by Rho family GTPases, LXR/RXR activation, hypertrophy of cardiac muscle, apoptosis of kidney cells, and necrosis of liver. On the other hand, time (24, 48 and 96h) exhibited higher similarity than oil types, suggesting the pathway profiles might be more time-dependent than oil-type-dependent. The lists of DEGs responsible for the enrichment canonical pathways are reported in Table S4.

**Figure 4:**
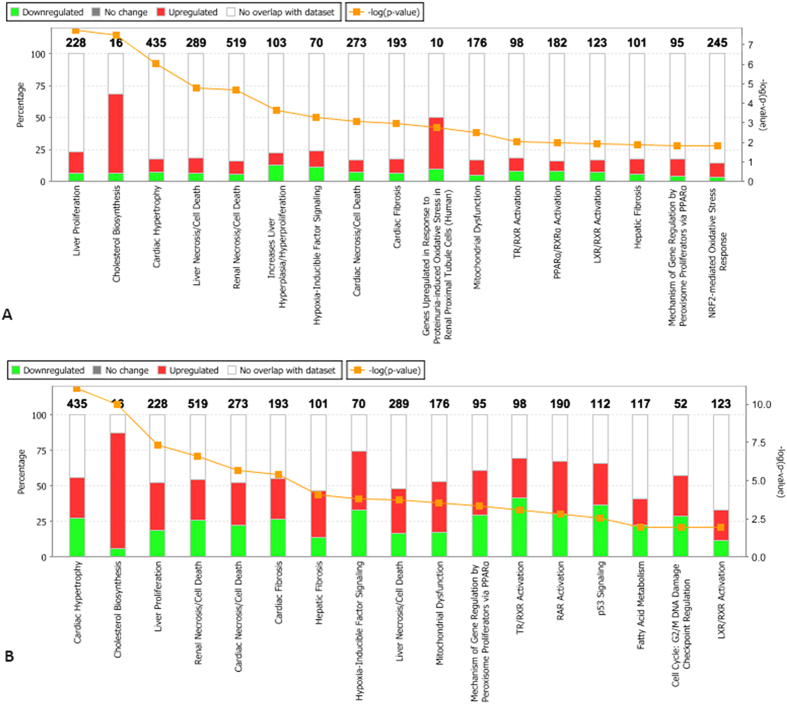
Stacked Bar Charts display the number of up-regulated (red), down-regulated (green) in each tox-pathway as a percentage of the total number of dataset molecules overlapping with the Tox Lists by *de novo* (**A**) and OnRamp (**B**) approaches.

**Figure 5:**
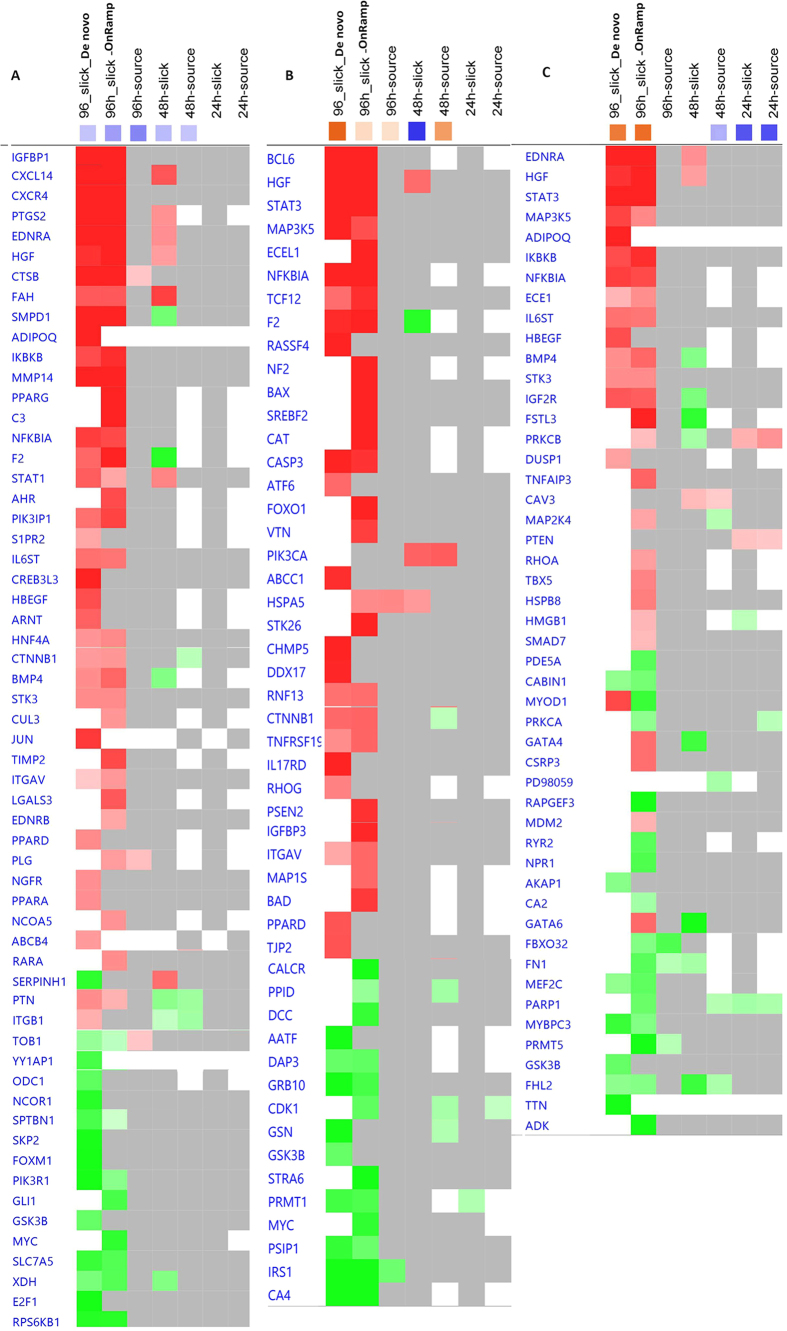
Heatmaps of genes in inhibited proliferation of liver cells (**A**), apoptosis of kidney cells (**B**) and hypertrophy of cardiac muscle (**C**) across different transcriptome analytic approaches, oil types and time. Heatmap colors: Red, up-regulated in the dataset; Green, down-regulated in the dataset; Gray; in the dataset but not analysis-ready (i.e. did not pass cutoffs and filters); White, not in the dataset. The orange or blue-colored squares in the column headers indicate the z-score (activity prediction) for each analysis. Orange shading indicates predicted activation and blue shading indicates predicted inhibition.

Furthermore, we also examined toxicity pathways which were enriched using the IPA-Tox tool (p < 0.05), focusing toxicity assessment of input molecules using toxicogenomic approaches. The toxicities of the slick oil-interacting DEGs identified by *de novo* and OnRamp approaches were analyzed and compared to each other. The two approaches shared 20 common toxicity pathways among the top 30 significant ones. Liver proliferation (p-value: 1.84E-08), cholesterol biosynthesis (p-value: 3.17E-08) and cardiac hypertrophy (p-value: 9.52E-07) topped the list generated from the *de novo* approach, while cardiac hypertrophy (p-value: 9.01E-12), cholesterol biosynthesis (p-value: 9.85E-11) and liver proliferation (p-value: 4.85E-08) topped the list from OnRamp (Fig. 4). The DEGs responsible for liver proliferation, cardiac hypertrophy and apoptosis of kidney were identified, and the common DEGs may be used as biomarkers for detecting DHW oil toxicity (Fig. 5; Table S5). Again, the 96h_slick_*De novo* and the 96h_slick_OnRamp showed the highest similarity of DEG profiles in each tox function. A full list of toxicity-pathways and DEGs by IPA-Tox are provided in Table S6.

## Discussion

The effects of crude oil on fish development have been well established using conventional morphological assessment. However, morphological evaluation can be subjective and subtle/delayed effects (such as delayed mortality or reduced swimming performance) may remain unnoticed. Moreover, classic morphological assessment using microscopy is relatively labor intensive and therefore a challenge for large samples during rapid embryonic development^17^. Global gene expression using transcriptomic tools can aid in detecting subsequent phenotypic effects and also anchoring multiple morphological impacts at one time of assessment, which may improve predictability, sensitivity and efficiency of the toxicity assessment. Yang^19^ revealed barcode-like toxicogenomic responses in zebrafish embryos exposed to eleven compounds prior to the onset of phenotypic effects. A recent study in mahi-mahi focused on the developmental cardiotoxicity of crude oil exposure and targeted ten molecular indicators of cardiac stress and suggested the necessity to design species-specific molecular markers^17^. To our knowledge, the present study is the first to *de novo* assemble the mahi-mahi transcriptome. An extensive list of phenotype-associated gene markers for mahi-mahi was identified from commonly differentially expressed genes between different oil types and different developmental stages. This comparative DEG analysis illustrated similarity and differences in responses to different oil types at different developmental times at individual gene levels. However, it is reasonable to expect that the numbers of common DEGs used for biomarkers will decrease when more developmental stages, more oil types, more concentrations of oil, and more species are included. Therefore, comparative pathway analysis was also employed in the present study. Similar to previous studies on embryonic stem cell^20^ and zebrafish embryo^21^, we found that whereas individual genes within a pathway were not significantly altered, significant activation/inhibition of common toxicity pathways still occurred. This comparative toxicity-pathway analysis also provided additional information on modes of action of crude oil, closely relating to the concept of the adverse outcome pathway^22^. Moreover, as most enriched GO and proposed pathways were from vertebrates, notably humans, it may also enable an extrapolation of crude oil toxicity to the human situation.

**Figure 6:**
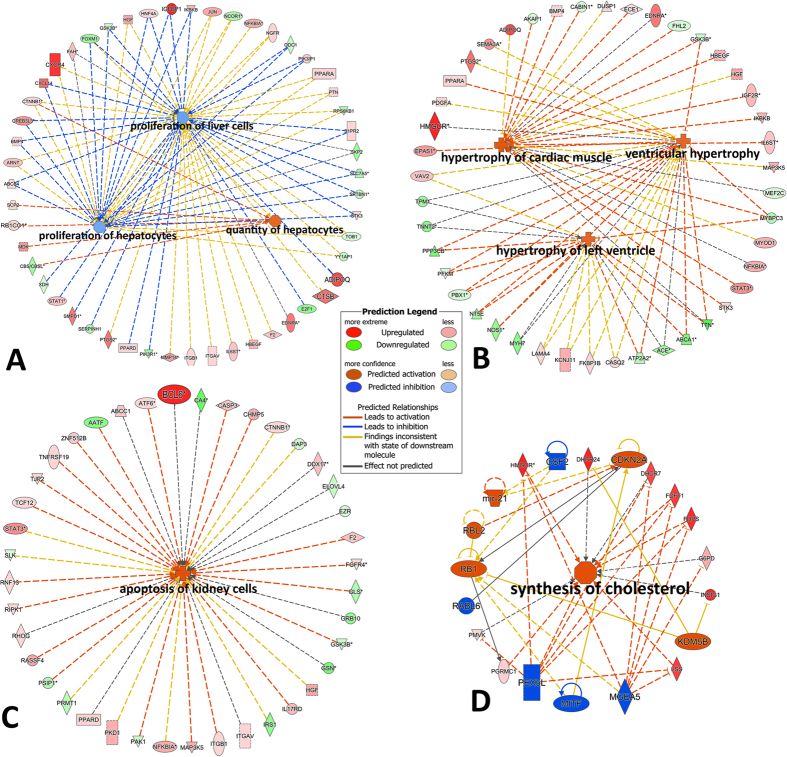
Top significant toxicity pathways through IPA-Tox showing how slick oil may impact/lead to (**A**) inhibition of liver proliferation; (**B**) cardiac hypertrophy; (**C**) apoptosis of kidney cells; (**D**) cholesterol biosynthesis.

One of our objectives was to determine whether there were commonalities in the gene expression profiles generated by the Trinity *de novo* approach and the reference-transcriptome-guided automatic OnRamp approach. The results indicated a large proportion of DEGs, and GO terms common to both approaches, although OnRamp exhibited relatively more extensive changes (more enriched DEGs, GOs and pathways). We found that whereas individual genes within a gene pathway were not significantly regulated, significant common regulation at pathway categories still occurred. In this regard, we explored and focused on toxicity pathways, which were not limited by differences on the level of individual gene expression. Given that DWH oil is a complex chemical mixture, developmental toxicity is likely to arise from multiple mechanisms and pathways. IPA-Tox examination pointed to high commonalities between the two approaches, reflecting highly similar transcriptional responses to slick oil toxicity. The following discussion emphasizes the most significant enriched toxicity pathways and their responsive genes that were commonly observed in the two approaches, including liver proliferation, cholesterol biosynthesis, cardiac hypertrophy, renal necrosis/apoptosis, TR/RXR and LXR/RXR activation (Fig. 6).

Liver proliferation, the highest enriched toxicity function (Fig. 4A), occurs in response to partial hepatectomy and also after chemical injury^23^. Exposure to benzo[a]pyrene (BaP), a model polycyclic aromatic hydrocarbon (PAH), leads to increased liver tumorigenesis^24^ and adiposity as well as hepatic steatosis^25^ in animals. Liver proliferation was identified in the top three most significantly affected mechanistic responses in Atlantic cod (*Gadus morhua*) larvae exposed to mechanically dispersed oil for four days^26^. Liver proliferation is associated with signaling cascades involving growth factors, cytokines, hormones, nuclear receptors and alterations of growth related signals^27^. The changes of 55 interacting molecules were predicted to affect liver proliferation by IPA (Fig. 6A). Significant upregulation of *cxcl14, adipoq, bmp4, ppard*, and *pik3ip* genes may be associated with inhibited growth and proliferation of liver in mice. For example, *cxcl14* over-expression aggravated CCl_4_-induced liver injuries, evidenced by enhanced acidophilic change and necrosis as well as increased fat deposition in hepatocytes, resulting in inhibited hepatocyte proliferation^28^. Adiponectin (*adipoq*) decreases proliferation of activated rat hepatic stellate cells^29^. In mouse, homozygous mutant mouse peroxisome proliferator activated receptor delta (*ppard* gene knockout) increases proliferation of mouse hepatic stellate cells in cell culture^30^, and a negative regulator of PI3K (*pik3ip1*) decreases proliferation of hepatocytes in liver^31^. More recently, Do^32^ found bone morphogenetic protein 4 gene (*bmp4*) inhibits proliferation of primary hepatocytes and HepG2 cells in culture. Work on zebrafish hepatogenesis has revealed that similar genes regulate hepatic patterning in both fish and mice^33^. The ability for the liver to regenerate is central to liver homeostasis. Because the liver is the main site of chemical detoxification, it is exposed to many chemicals (oil-derived PAHs in this case) in the body, which may potentially induce injury in cells other than hepatocytes. It is possible that the DHW oil exposure inhibits proliferation of damaged hepatocytes (as predicted by IPA in Fig. 6A) thereby diminishing detoxification and recovery.

**Figure 7:**
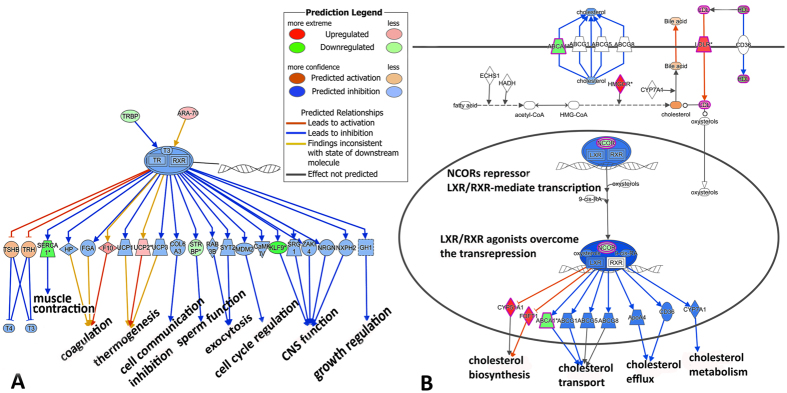
A series of functions (e.g. muscle contraction, cell cycle regulation, CNS funtion, cholesterol biosynthesis and metabolism) pertubated through TR/RXR (**A**) and LXR/RXR (**B**) pathways predicted by IPA.

Slick oil had a profound impact on the expression of genes involved in cholesterol biosynthesis in mahi-mahi larvae as indicated by both GO and pathway analyses (Figs S2 and 6). Consistent with our mahi-mahi data, Olsvik^26^ identified cholesterol biosynthesis as one of the five most significant impacted pathways in Altantic cod *Gadus morhua* larvae exposed to chemically and mechanically dispersed oil. Cholesterol is a major structural component of the plasma membrane of eukaryotes and determines important biophysical characteristics of the cell surface bilayer. Membrane cholesterol dynamics also plays a key role in other cellular processes, such as intracellular sorting and vesicle transport. Due to the importance of cholesterol in these functions, cholesterol concentration within the cell is tightly regulated^34^. Cholesterol is either derived from the diet or from *de novo* synthesis occurring mainly in the liver through the mevalonate pathway. This pathway comprises several critical enzymes such as 3-hydroxy-3-methylglutaryl-coenzyme A reductase (*hmgcr*), farnesyl diphosphate synthase (*fdps*) farnesyl-diphosphate farnesyltransferase (*fdft1*), and 2,3-oxidosqualene-lanosterol cyclase (*lss*) and 7-dehydrocholesterol reductase (*dhcr7*)^35^,^36^,^37^,^38^,^39^. All of these genes were significantly downregulated in 96hpf mahi-mahi larvae by slick oil exposure (Fig. 6D). The inhibition of cholesterol transport, efflux and metabolism also affects the LXR/RXR pathway which also regulates ATP binding cassette transporters (*abca1, abcg1, abcg5 and abcg8*), cytochrome P450 51A1 and 7A1^40^,^41^,^42^,^43^ (Fig. 7). Interestingly, Tanos^44^ established an important relationship between aryl hydrocarbon receptor (AHR) and cholesterol biosynthesis pathway independent of its dioxin response element (DRE) in both mouse liver and primary human hepatocytes. A coordinate repression of genes involved in cholesterol biosynthesis, namely *hmgcr, fdft1, sqle* and *lss*, and subsequently decreased cholesterol secretion were observed following AHR activation.

Approximately 80 genes differentially expressed at 96 hpf mahi-mahi larvae after slick oil exposure were involved in cardiac toxicity as determined by the *de novo* approach (Fig. 6B). The OnRamp approach also identified genes associated with cardiac toxicity as well as cardiac muscle development, contraction, K^+^ and Ca^2+^ homeostasis. For example, calsequestrin 2 (*casq2*), plays important roles in maintaining the cellular Ca^2+^ levels modulating contraction of cardiac muscle^45^, and was significantly upregulated. Bmp4 was also upregulated which is consistent with induced cardiomyocyte hypertrophy, apoptosis, and cardiac fibrosis^46^. Interestingly, pathological consequences were inhibited in mice treated with the bmp4 inhibitors noggin and DMH1^46^. Troponin T type 2 (*tnnt2*) was significantly downregulated 96 hpf after slick oil exposure, which would also result in changes of cardiac muscle contraction in response to alterations in intracellular calcium ion concentration^47^. The inhibition of muscle contraction can also be mediated through the thyroid receptor/retinoid receptor (TR/RXR) pathway (Fig. 7), or downregulation of sarcoplasmic-endoplasmic reticulum calcium ATPase gene (*serca1*)^48^. Each of these was diminished by oil exposure. Another mechanism of crude oil-caused cardiotoxicity is a blockade of the IKr potassium current, which drives the repolarization phase of the cardiac action potential^49^. We also found potassium voltage-dependent potassium channel genes (*kcnj11* and *kcnq1*) were significantly downregulated in 96 hpf after slick oil exposure. A recent study of Altantic haddock embryos exposed to crude oil also found a reduction of mRNA encoding cardiac ion channels^16^. In mahi-mahi, crude oil exposures significantly reduced the expression of genes related to cardiomyocyte contractility and cardiac defects, such as atrial and ventricular myosin heavy chain and cardiac myosin light chain genes^17^. In the present study, *myh6, myh7, myl4,* and *myl6* were significantly downregulated. Two sarcoplasmic-endoplasmic reticulum calcium ATPase genes (*serca1* and *sercal2*) and sodium/potassium/calcium exchanger (*nckx*) were also significantly downregulated. This suggests that slick oil can modify cardiac ion channels, pumps, and exchangers. The relationship between ion signaling and transcription of ion channel genes most likely reflects the recently characterized process of excitation-transcription (ET) coupling^50^. It was suggested that crude oil may act through a dual mechanism by blocking both ion channel function and also feedback to transcriptional regulation of ion channel genes^16^. These affected cardiac genes have been anchored to the higher order cardiac syndrome characteristics of crude oil exposure in mahi-mahi larvae (heart rate, pericardial and yolk sac edema)^10^.

In addition to cardiac toxicity, PAHs have been shown to cause significant renal toxicity in both freshwater and marine fish species. For example, Incardona^11^ have demonstrated that embryonic exposure to PAHs can cause primary effects on cardiac conduction that have secondary consequences for late stages of cardiac morphogenesis, kidney development, neural tube structure and formation of the craniofacial skeleton. Head kidney was also a sensitive target of exposure to DWH oil on resident gulf killifish (*Fundulus grandis*)^51^. Benzanthrone showed evidence of patchy glomerular congestion, tubular lesions, and damaged epithelial cells in kidney^52^. Incardona and Scholz^53^ suggested the most likely cause for edema in pink salmon larvae exposed to oil-derived PAHs was the inability of a failing heart and kidney. While PAHs are shown to cause nephrotoxicity and there are many know mechanisms of renal toxicity from membrane disruption to metabolic inhibition^54^, the underlying molecular mechanisms in fish are largely unknown. In the current study with mahi-mahi, renal necrosis/apoptosis was ranked No. 5 (p-value: 2.12E-05) and No. 4 (p-value: 3.69E-07) by *de novo* and OnRamp approaches, respectively (Fig. 4). The predicted induction of apoptosis of kidney cells are based on the expression profile of a number of important kinases, including mitogen-activated protein kinase (MAP3K)^55^, receptor interacting serine/threonine kinase (RIPK1)^56^, tight junction protein (TJP2)^57^, p21 protein (Cdc42/Rac)-activated kinase (PAK1)^58^, and phosphatidylinositol-4,5-bisphosphate 3-kinase (FGFR4)^59^ (Fig. 6C). Although functional effects of renal toxicity of crude oil have been rarely reported, it is reasonable to expect that exposure to crude oil that induce apoptosis in kidney may create a critical disturbance in kidney homeostasis, injury to renal tubular epithelial cells, and affect glomerular filtration, active secretion and urine formation, contributing to acute or chronic renal failure^54^,^60^,^61^.

In summary, the first *de novo* approach applied in this study, was used to determine the global transcriptomic responses of mahi-mahi larvae exposed to DWH oil. Compared with the reference-transcriptome-guided automated OnRamp approach, a large proportion of DEGs, GO terms and toxicity pathways were common, with genes involved in several toxicity-pathways (liver proliferation, cholesterol biosynthesis, cardiac hypertrophy, renal necrosis/apoptosis, TR/RXR and LXR/RXR activation). Genes involved in these pathways may serve as biomarkers to predict toxicities and/or recovery of DWH oil. There is a need to conduct focused follow-up studies to confirm the initial transcriptomic results with additional methods (e.g. dose-response assays) at an expanded range of oil concentrations in multiple fish species. Overall, the present study has provided valuable molecular resources for facilitating further investigations on the holistic understanding of the developmental toxicity of DWH oil in mahi-mahi. The developed *de novo* and comparative toxicity pathway methods will also be useful for toxicogenomic study on other non-model fish species.

## Methods

### Animals and DHW oil exposure

Mahi-mahi (*Coryphaena hippurus*) broodstock were caught off the coast of South Florida using hook and line angling techniques and then directly transferred to University of Miami Experimental Hatchery (UMEH). Broodstock were acclimated in 80 m^3^ fiberglass maturation tanks equipped with recirculating and temperature controlled water. All embryos used in the experiments described here were collected within 2–10 h following a volitional (non-induced) spawn using standard UMEH methods^62^. Two sources of crude oil from the DWH spill that varied with respect to the state of weathering were obtained from British Petroleum under chain of custody for testing purposes: (1) slick oil collected from surface skimming operations (sample ID: OFS-20100719-Juniper-001 A0091G) and (2) oil from the Massachusetts barge (sample ID: SO-20100815-Mass-001 A0075K) which received oil collected from the subsea containment system positioned directly over the well (referred to herein as slick and source oil, respectively). Preparation of water accommodated fractions (HEWAFs) of two oil types, and oil exposures were performed at the University of Miami as outlined previously^13^. Time course (24, 48 and 96 hpf) exposures were performed using the nominal LC25s determined from the aforementioned bioassays for RNA sequencing. The LC25 concentration (2% HEWAF of slick oil or 0.09% HEWAF of source oil) was chosen as a compromise between attempting to capture initiating events as well as significant cascade effects while ensuring that a sufficient signal was observed. Three replicates were used per time point with 25 embryos per replicate. Slick and source oil HEWAF exposures were run concurrently using the same batch of embryos and a shared set of controls for both sets of experiments. A full description of the samples collection and exposure is presented in Xu^10^. All animal experiments were performed ethically and in accordance with Institutional Animal Care and Use Committee (IACUC protocol number 15–019) approved by the University of Miami IACUC committee, and the institutional assurance number is A-3224–01.

### RNA isolation, cDNA library construction and sequencing

The surviving embryos or larvae from each replicate were pooled and RNA was isolated and purified with RNeasy Mini Kit (Qiagen, Valencia, California). The total RNA sample was quantified by NanoDrop ND-1000 Spectrophotometer (Nanodrop Technologies, Wilmington, DE, USA). 200 ng of total RNA was used to prepare RNA-Seq libraries using the TruSeq RNA Sample Prep kit following the protocol described by the manufacturer (Illumina, San Diego, CA). Single Read 1 × 50 sequencing was performed on an Illumina HiSeq 2500 at the Center for Genomics Medicine, Medical University of South Carolina, Charleston, SC, with each individual sample sequenced to a minimum depth of ~50 million reads. Data were subjected to Illumina quality control (QC) procedures (>80% of the data yielded a Phred score of 30). A detailed description of these methods is presented in Xu^10^. The read data for the 32 samples was deposited in the NCBI database (Accession Number: GSE79675).

### *De novo* assembly and annotation of the Mahi-mahi transcriptome: *de novo* approach

Raw sequences were trimmed off Illumina adapter sequences and filtered out sequences that did not meet the quality thresholds using Trimmomatic (version 0.33)^63^. All reads were pooled and assembled *de novo* using Trinity (version 2.2.0)^64^ with k-mer length set at 25. According to Grabherr^64^, Trinity is a *de novo* algorithm recently developed for reconstructing transcriptome using de Bruijn graphs. Transcriptome assembly is challenging mainly due to the unevenly distributed RNA-Seq data coverage and alternative splicing in individual genes. Trinity consists of three parts: Inchworm, Chrysalis, and Butterfly. Trinity firstly makes linear contigs from RNA-Seq reads, and Chrysalis, the second step of Trinity, clusters minimally overlapping Inchworm contigs into sets of connected components that comprise alternative splice forms or closely related paralogs and then generates and expands de Bruijn graphs, and finally outputs the transcripts as well as isoforms. The process starts by decomposing the reads into small overlapping k-mers and extending them by coverage. Alternative splicing is determined by finding the common sections of the intermediate transcripts, and those transcripts are re-grouped. The de Bruijn graphs are generated by integration of isoforms that are similar except one base. And finally, by finding and expanding the common section of transcripts and representing the most compact path on the graph, Trinity delivers the fully assembled transcriptome^64^. Subsequently, the high quality reads from 96 hpf larvae samples (slick oil treatments and controls) were mapped to the Trinity assembled *de novo* mahi-mahi transcriptome to estimate the transcript/unigene abundance using RSEM (RNA-Seq by Expectation-Maximization) (version 1.2.21)^65^ with the default aligner Bowtie (version 1.1.1)^66^. The expression level of each transcript/Unigene was measured with FPKM (fragments per kilobase per million reads). A script ‘PtR’ in Trinity was used to compare the biological replicates for each of the samples and generate a correlation matrix and Principal Component Analysis (PCA) plot. Subsequent analyses in RSEM were conducted using a selection of scripts provided as part of the Trinity package (version 2.2.0). Statistical differences in gene expression levels between 96 hpf mahi larvae exposed to slick oil and controls were calculated using DEseq2. Genes were considered differentially expressed when false discovery rate (FDR) < 0.05 (Benjamini−Hochberg correction). Trans-decoder (version 3.0.0) was used for coding sequence prediction, and Trinotate (2.0.2) for functional annotation. Trinotate is a comprehensive annotation suite specifically designed for automatic functional annotation of *de novo* assembled transcriptomes of non-model organisms, including homology search to known sequence data (BLAST + /SwissProt), protein domain identification (HMMER/PFAM), protein signal peptide and transmembrane domain prediction (signalP/tmHMM), and leveraging various annotation databases (eggNOG/GO/Kegg databases), reporting the best hits in the databases (http://trinotate.github.io/). All analysis was carried out on a local server running under the Institute for Integrative Genome Biology (IIGB)‘s Linux cluster, Biocluster, environment (http://manuals.bioinformatics.ucr.edu/home/hpc). The overall workflow is summarized graphically in Fig. 1.

### A reference-transcriptome-guided analysis by OnRamp: OnRamp approach

A secondary analysis was carried out on an OnRamp Bioinformatics Genomics Research Platform (OnRamp Bioinformatics, San Diego, CA) as previously described^10^. OnRamp’s advanced Genomics Analysis Engine utilized an automated RNAseq workflow to map read alignment to the *Takifugu rubripes* transcriptome (FUGU4) using BLASTX: Basic Local Alignment Search Tool, generate gene-level count data, and differential expression analysis with DEseq2. Transcript count data from DESeq2 analysis of the samples were sorted according to their false discovery rate (FDR) at which a transcript is called significant. The protein FASTA sequences from Ensembl for *Fugu* were compared using Ensembl’s homology to create protein FASTA files that contained a human Entrez gene ID that mapped via *Fugu* to *Mahi-mahi*. A detailed description of these methods is presented in Xu^10^.

### Gene ontology and Ingenuity Pathway Analyses

The two sorted transcript lists of mahi generated by both *de novo* and OnRamp approaches were mapped to human orthologs to generate HGNC (HUGO Gene Nomenclature Committee) gene symbols for downstream gene ontology (GO) term analysis, using Gorilla^67^ and ToppGene Suite^68^. This approach has been demonstrated to improve functional analysis of fish genes with a more sensitive systems level interrogation, by providing access to the best-annotated databases and tools for human/mouse/rat models^10^,^69^,^70^, while limitations of the mapping due to the extra genome duplication events in teleost fish and species differences in gene functions still exist. GO terms for molecular function, molecular component, biological process, pathway and phenotype were considered significantly enriched when p < 0.05. Considering gene duplication in fish species and the possible functionally different homologues between fish and human/mouse/rat^71^,^72^, the same set of the *de novo* transcript lists were also examined using the enrichment of GO terms against the zebrafish (*Danio rerio*) reference using DAVID Bioinformatics Resources 6.8^73^. The analysis was performed on categories for biological process (GOTERM_BP_FAT), cellular component (GOTERM_CC_FAT) and molecular function (GOTERM_MF_FAT) using the functional annotation tool with a modified Fisher exact p-value (EASE score) < 0.01.

Comparative toxicity pathway (tox-pathway) analysis was further conducted. If individual genes are not commonly regulated between methods/conditions, commonality may still exist on the level of pathway regulation. Comparative pathway analysis could also provide additional information on modes of action of toxicants. This comparative toxicity pathway analysis closely relates to the concept of adverse outcome pathways determining toxicity^22^. Ingenuity Pathway Analyses (IPA) (Ingenuity Systems Inc., Redwood City, CA, USA) was used to compare *de novo* and OnRamp approaches at different oil types and developmental time conditions to identify similarities and differences in canonical pathways and toxicity functions (IPA-Comparison analysis). IPA-Tox is an integral component of an integrated systems toxicology approach specifically for toxicity assessment. This component of IPA is taken from peer-review literature and manually reviewed by experts in the field of toxicology (both traditional and toxicogenomics), drawing on a library of hundreds of signaling and metabolic pathways. It provides pathway content as well as gene to phenotype associations from toxicity studies. By linking DEGs to their known role in specific tox endpoints, analysis of toxicogenomics data in IPA-Tox identifies subsets of DEGs that may be predictive of certain toxicity endpoints. IPA-Tox was used to examine the toxicity pathways and reveal biological pathways/mechanisms underlying toxicity-specific phenotypes, as well as to provide insights into candidate phenotype-specific biomarkers. Fisher’s exact test was used to calculate a p-value determining the probability that the association between the genes in the dataset and the IPA-Tox pathways as opposed to this occurring by chance alone.

## Additional Information

**How to cite this article:** Xu, E. G. *et al*. Novel transcriptome assembly and comparative toxicity pathway analysis in mahi-mahi (*Coryphaena hippurus*) embryos and larvae exposed to Deepwater Horizon oil. *Sci. Rep.* **7**, 44546; doi: 10.1038/srep44546 (2017).

**Publisher's note:** Springer Nature remains neutral with regard to jurisdictional claims in published maps and institutional affiliations.

## Supplementary Material

Supplementary Information

## Figures and Tables

**Table 1:** Summary statics of mahi-mahi transcriptome assembly and annotation.

Total assembled bases	42,447,657
Total trinity transcript contigs	60,842
Percent GC	49.6
Transcrip contig N10	3,201
Transcrip contig N20	2,348
Transcrip contig N30	1,818
Transcrip contig N40	1,417
Transcrip contig N50	1,093
Median contig length	419
Average contig	711
BLASTX_hit	30,518
BLASTP_hit	22,302
Pfam	17,370
ThHMM	3,168
eggnog	26,444
GO_blast	29,007
GO_pfam	11,401
