# Supplementary material for: Novel transcriptome assembly and comparative toxicity pathway analysis in mahi-mahi (Coryphaena hippurus) embryos and larvae exposed to Deepwater Horizon oil
Source: Sci Rep. 2017 Mar 15;7:44546. doi: 10.1038/srep44546 (PMC5353654; doi:10.1038/srep44546)
Supplement: Supplementary Information [file srep44546-s1.pdf]

Supplementary Information

Novel transcriptome assembly and comparative toxicity pathway analysis in mahi-mahi (*Coryphaena hippurus*) embryos and larvae exposed to Deepwater Horizon oil

Elvis Genbo Xu[1*], Edward M. Mager[2], Martin Grosell[3], E.Starr Hazard[4,5], Gary Hardiman[4,6,7], Daniel Schlenk[1]

[1] Department of Environmental Sciences, University of California, Riverside, CA 92521

[2] Department of Biological Sciences, University of North Texas, Denton, TX 76203

[3]Department of Marine Biology and Ecology, University of Miami, Miami, FL 33149

[4] Center for Genomics Medicine, Medical University of South Carolina, Charleston, SC 29403

[5] Computational Biology Resource Center, Medical University of South Carolina, Charleston, SC 29403

[6] Departments of Medicine & Public Health Sciences, Medical University of South Carolina, Charleston, SC 29403

[7] Laboratory for Marine Systems Biology, Hollings Marine Laboratory, Charleston, SC 29412

*Corresponding author

Corresponding author: Elvis Genbo Xu

Corresponding email: genboxu@ucr.edu

Corresponding address: Department of Environment Sciences, University of California, Riverside, CA 92521, USA

Corresponding Tel.: 1-951-313-7643

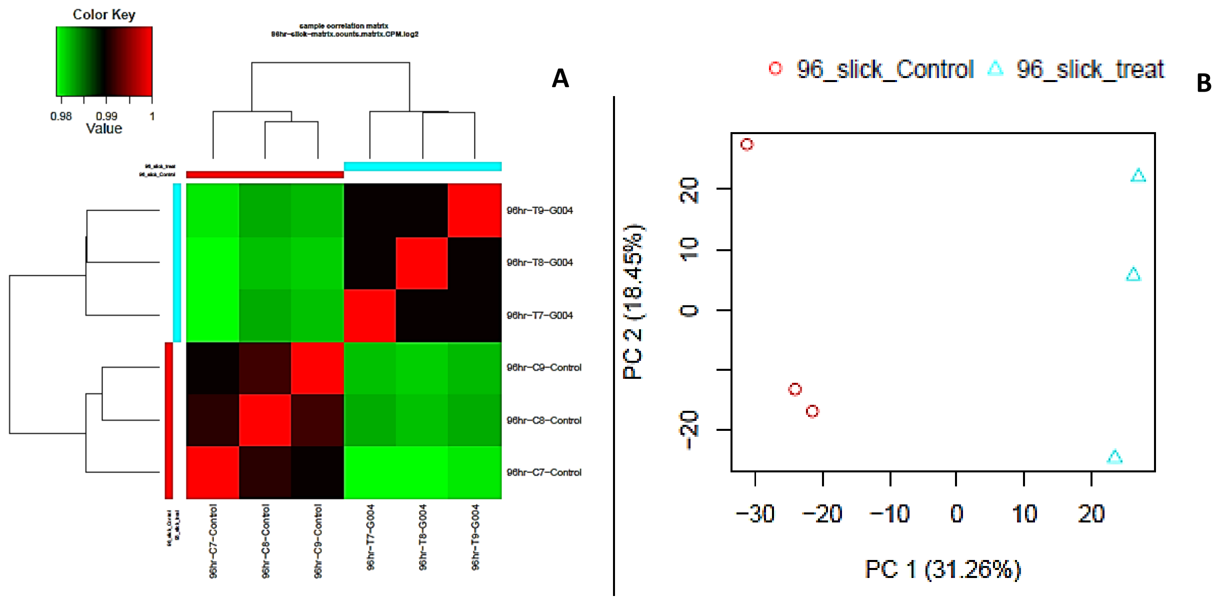

Fig. S1 Heatmap (a) showing the Euclidean distances between the samples as calculated from the DEseq2 variance stabilizing transformation of the count data. Samples are clustered by similarity. The samples from each treatment cluster together indicating global differences between the slick oil treatment and control. PCA plot (b) indicating that the first principal component (31.26%) separates the 96 hpf control and slick oil samples into the two sides of x = 0, indicating that the two groups are different.

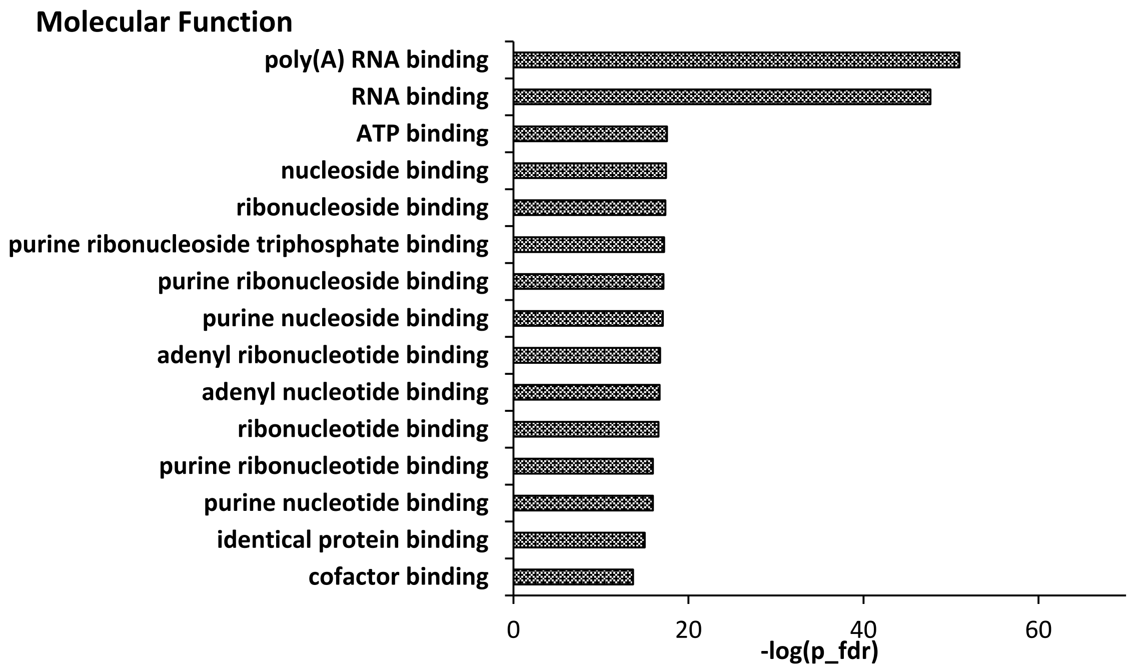
Molecular Function
poly(A) RNA binding
RNA binding
ATP binding
nucleoside binding
ribonucleoside binding
purine ribonucleoside triphosphate binding
purine ribonucleoside binding
purine nucleoside binding
adenyl ribonucleotide binding
adenyl nucleotide binding
ribonucleotide binding
purine ribonucleotide binding
purine nucleotide binding
identical protein binding
cofactor binding
0
20
40
60
-log(p_fdr)

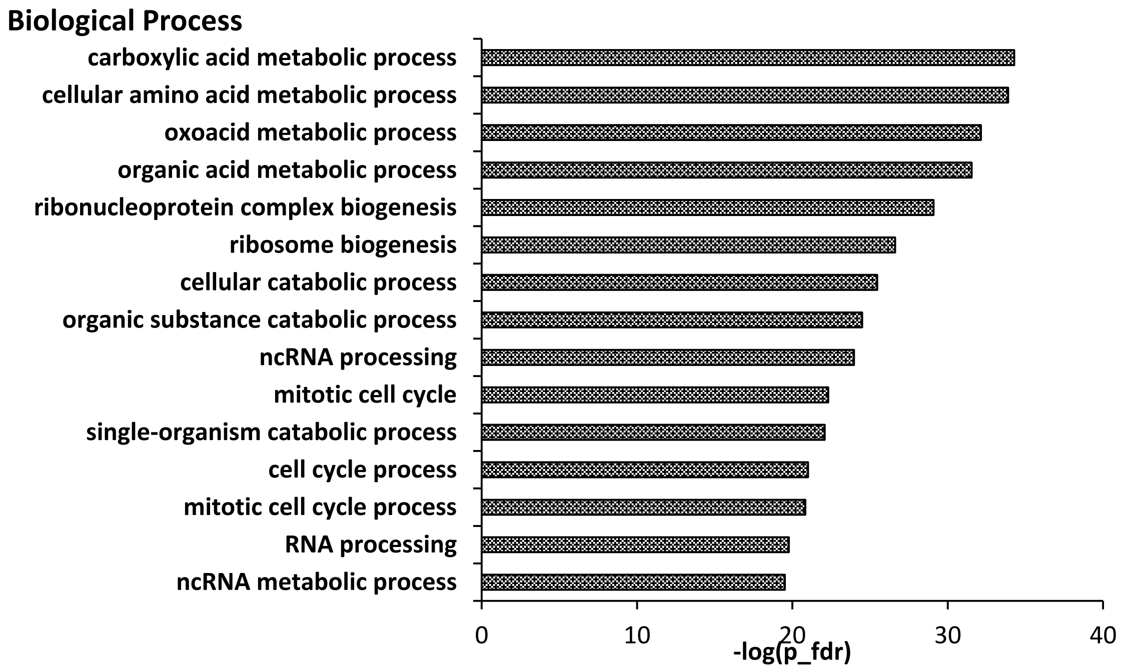
Biological Process
carboxylic acid metabolic process
cellular amino acid metabolic process
oxoacid metabolic process
organic acid metabolic process
ribonucleoprotein complex biogenesis
ribosome biogenesis
cellular catabolic process
organic substance catabolic process
ncRNA processing
mitotic cell cycle
single-organism catabolic process
cell cycle process
mitotic cell cycle process
RNA processing
ncRNA metabolic process
0
10
20
30
40
-log(p_fdr)

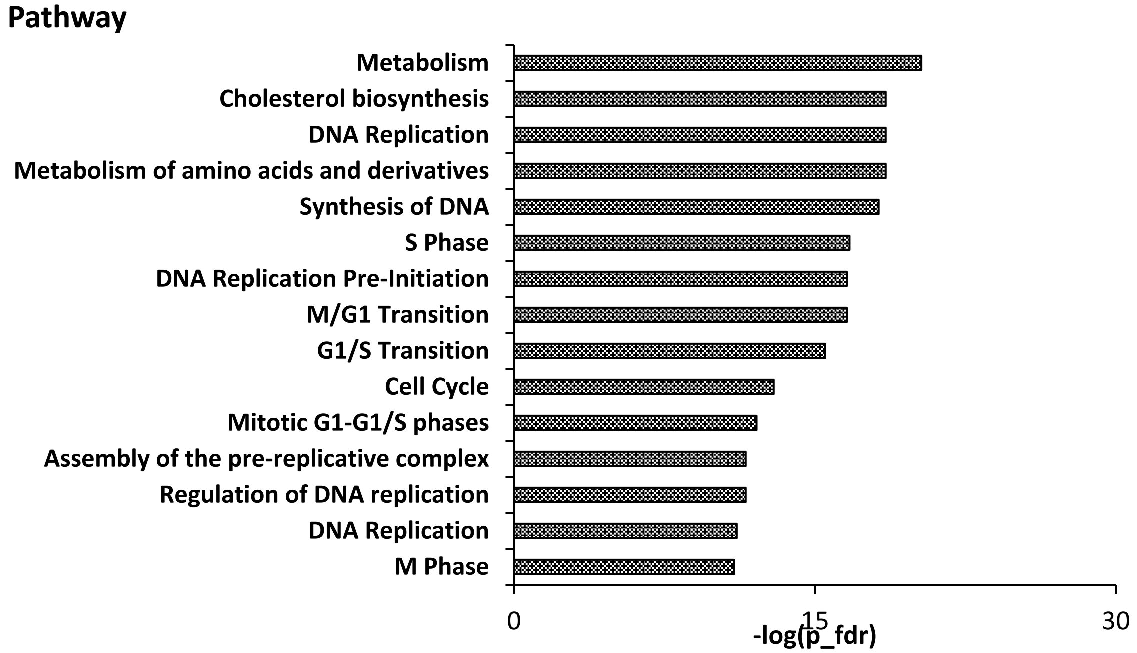

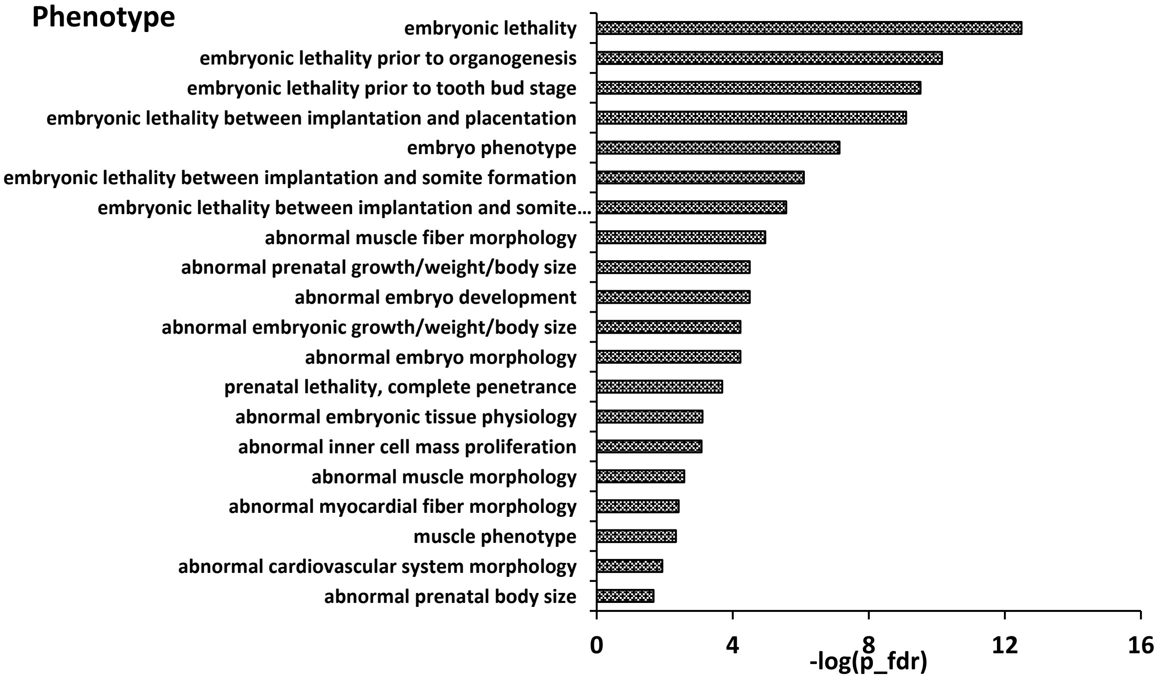

Fig. S2 Enriched molecular function, biological process, pathway and phenotype by *De novo* approach. 96 hpf mahi-mahi larvae were exposed to slick oil. The X-axis is a negative log of the adjusted p-value.

Table S1 Top enriched molecular functions, biological processes and cellular components by GOrilla.

De novo			OnRamp		
**Molecular Functions**	**q_FDR**	**Gene**	**Molecular Functions**	**q_FDR**	**Gene**
oxidoreductase activity	4.33E-07	57	oxidoreductase activity	3.46E-12	103
antioxidant activity	8.11E-06	14	cofactor binding	6.21E-09	34
lipase activity	9.64E-06	15	structural constituent of eye lens	3.36E-07	7
receptor activity	3.04E-05	48	catalytic activity	1.35E-06	120
molecular transducer activity	3.04E-05	48	coenzyme binding	1.37E-06	18
oxidoreductase activity, acting on a [illegible]	5.78E-05	4	ion binding	2.18E-06	296
carboxylic ester hydrolase activity	9.07E-05	18	cation binding	4.43E-06	282
oxidoreductase activity, acting on paired donorsration	1.26E-04	3	metal ion binding	5.21E-06	279
tetrapyrrole binding	2.08E-04	13	iron ion binding	6.26E-06	27
heme binding	2.41E-04	9	oxidoreductase activity, acting on paired	1.03E-05	4
flavonoid 3'-monooxygenase activity	3.34E-04	1	oxidoreductase activity, acting on the CH-	1.19E-05	5
oxidoreductase activity, acting on	3.34E-04	1	aminopeptidase activity	1.51E-05	13
vitamin D 24-hydroxylase activity	3.34E-04	1	monooxygenase activity	8.79E-05	6
serine-type peptidase activity	3.35E-04	18	heme binding	9.25E-05	17
serine hydrolase activity	3.35E-04	18	oxygen binding	1.35E-04	7
transmembrane receptor activity	3.55E-04	31	oxidoreductase activity, acting on paired	1.66E-04	24
monooxygenase activity	5.08E-04	3	sulfur compound binding	1.71E-04	25
transmembrane signaling receptor	6.15E-04	29	oxidoreductase activity, acting on the	1.80E-04	6
oxidoreductase activity, acting on the	7.57E-04	3	tetrapyrrole binding	1.98E-04	17
serine-type endopeptidase activity	8.26E-04	15	oxidoreductase activity, acting on the	2.49E-04	5
**Biological Processes**	**q_FDR**	**Gene**	**Biological Processes**	**q_FDR**	**Gene**
cholesterol biosynthetic process	1.56E-18	14	small molecule metabolic process	4.00E-16	106
secondary alcohol biosynthetic process	7.82E-19	14	single-organism metabolic process	4.25E-14	226
sterol biosynthetic process	5.21E-19	14	sterol biosynthetic process	8.25E-11	20
alcohol biosynthetic process	3.88E-17	14	cholesterol biosynthetic process	8.23E-11	12
steroid biosynthetic process	1.37E-16	14	secondary alcohol biosynthetic process	6.58E-11	12
cholesterol metabolic process	2.30E-16	16	sterol metabolic process	1.47E-10	26
secondary alcohol metabolic process	3.41E-16	16	oxidation-reduction process	1.55E-10	126
sterol metabolic process	5.05E-16	16	small molecule biosynthetic process	1.63E-10	40
steroid metabolic process	7.69E-16	17	alcohol biosynthetic process	2.00E-10	12
organic hydroxy compound [illegible]	2.77E-15	14	single-organism biosynthetic process	2.09E-10	61
lipid metabolic process	4.75E-13	112	steroid metabolic process	4.49E-10	29
alcohol metabolic process	5.99E-12	16	secondary alcohol metabolic process	7.07E-10	24
organic hydroxy compound metabolic	1.29E-11	16	cholesterol metabolic process	6.52E-10	24
small molecule biosynthetic process	2.65E-10	16	organic hydroxy compound metabolic	6.34E-10	36
lipid biosynthetic process	7.28E-09	15	alcohol metabolic process	6.08E-10	31
cellular lipid metabolic process	4.66E-08	87	steroid biosynthetic process	7.00E-10	12
single-organism metabolic process	1.85E-06	231	sensory perception of light stimulus	1.05E-09	26
single-organism biosynthetic process	3.61E-06	17	visual perception	9.96E-10	26

cholesterol biosynthetic process via	1.29E-05	4	organic acid metabolic process	1.79E-09	126
cholesterol biosynthetic process via lathosterol	1.23E-05	4	organic hydroxy compound biosynthetic process	1.91E-09	22
**Components**	**q_FDR**	**Gene**	**Components**	**q_FDR**	**Gene**
integral component of membrane	1.48E-13	238	extracellular region part	1.31E-10	363
intrinsic component of membrane	1.99E-13	241	extracellular region	6.16E-09	86
membrane part	1.21E-12	344	extracellular organelle	2.63E-08	288
vacuolar part	5.02E-09	101	extracellular vesicle	1.97E-08	288
vacuole	1.46E-08	79	extracellular exosome	2.82E-08	286
vacuolar lumen	5.61E-08	33	fibrillar collagen trimer	3.47E-08	10
lytic vacuole	1.50E-07	37	membrane-enclosed lumen	1.08E-07	53
lysosome	1.32E-07	37	organelle lumen	1.78E-07	51
lysosomal lumen	1.68E-07	30	extracellular space	1.91E-06	115
extracellular region part	1.72E-07	281	intracellular organelle lumen	2.60E-06	46
endoplasmic reticulum membrane	3.33E-06	22	membrane-bounded vesicle	4.24E-06	315
extracellular space	5.63E-06	71	vesicle	5.95E-06	324
plasma membrane	8.57E-05	204	collagen trimer	4.96E-04	13
extracellular exosome	2.25E-04	284	endoplasmic reticulum part	9.73E-04	47
endoplasmic reticulum	2.58E-04	37	endoplasmic reticulum membrane	2.16E-03	24
extracellular organelle	3.21E-04	284	cytoplasmic part	4.39E-03	612
extracellular vesicle	3.02E-04	284	ciliary membrane	1.12E-02	5
vacuolar membrane	2.92E-04	77	ciliary part	1.18E-02	6
plasma membrane part	3.12E-04	145	preribosome	1.44E-02	16
endoplasmic reticulum part	5.79E-04	22	extracellular matrix component	1.49E-02	22

Table S2 Top enriched molecular functions, components and biological processes by ToppGene

**De novo**			**OnRamp**		
**Molecular Functions**	**q_FDR**	**Genes**	**Molecular Functions**	**q_FDR**	**Genes**
poly(A) RNA binding	1.10E-51	404	poly(A) RNA binding	2.68E-27	338
RNA binding	2.26E-48	498	RNA binding	6.66E-24	412
ATP binding	2.86E-18	377	adenyl nucleotide binding	3.33E-16	378
nucleoside binding	3.78E-18	448	adenyl ribonucleotide binding	9.86E-16	372
ribonucleoside binding	4.38E-18	446	ATP binding	4.67E-15	361
purine ribonucleoside triphosphate	6.09E-18	442	purine nucleotide binding	1.79E-14	436
purine ribonucleoside binding	7.15E-18	444	ribonucleotide binding	3.71E-14	433
purine nucleoside binding	8.79E-18	444	purine ribonucleotide binding	9.11E-14	428
adenyl ribonucleotide binding	1.75E-17	381	nucleoside binding	1.13E-12	419
adenyl nucleotide binding	2.00E-17	383	ribonucleoside binding	1.13E-12	417
ribonucleotide binding	2.71E-17	451	purine ribonucleoside triphosphate	2.54E-12	412
purine nucleotide binding	1.17E-16	448	purine ribonucleoside binding	2.91E-12	414
purine ribonucleotide binding	1.17E-16	445	purine nucleoside binding	3.55E-12	414
identical protein binding	9.97E-16	342	protein complex binding	1.14E-11	237
cofactor binding	2.20E-14	103	structural molecule activity	2.78E-10	174

pyrophosphatase activity	1.55E-12	222	enzyme binding	1.70E-09	335
hydrolase activity, acting on acid	1.94E-12	222	cofactor binding	2.84E-09	92
hydrolase activity, acting on acid	2.12E-12	222	peptidase activity, acting on L-amino acid	9.11E-09	160
nucleoside-triphosphatase activity	3.04E-12	212	identical protein binding	9.79E-09	277
enzyme binding	7.89E-12	435	peptidase activity	3.01E-08	162
**Biological Processes**	**q_FDR**	**Genes**	**Biological Processes**	**q_FDR**	**Genes**
carboxylic acid metabolic process	5.29E-35	327	carboxylic acid metabolic process	7.20E-25	300
cellular amino acid metabolic process	1.29E-34	175	organic acid metabolic process	9.64E-24	325
oxoacid metabolic process	7.39E-33	346	oxoacid metabolic process	9.64E-24	321
organic acid metabolic process	2.91E-32	348	cellular component disassembly	4.36E-22	167
ribonucleoprotein complex	8.15E-30	184	tissue development	1.87E-20	445
ribosome biogenesis	2.46E-27	140	macromolecule catabolic process	6.93E-17	286
cellular catabolic process	3.47E-26	477	single-organism catabolic process	3.35E-16	458
organic substance catabolic process	3.26E-25	490	macromolecular complex subunit organization	1.73E-15	417
ncRNA processing	1.08E-24	156	cell development	3.49E-15	457
mitotic cell cycle	4.94E-23	297	organ morphogenesis	3.49E-15	250
single-organism catabolic process	8.28E-23	320	translational elongation	6.02E-15	64
cell cycle process	9.83E-22	371	cellular amino acid metabolic process	9.39E-15	155
mitotic cell cycle process	1.46E-21	274	mRNA metabolic process	1.45E-14	191
RNA processing	1.68E-20	267	response to endogenous stimulus	2.35E-14	348
ncRNA metabolic process	3.05E-20	187	extracellular matrix organization	4.27E-14	129
rRNA processing	6.82E-19	108	extracellular structure organization	5.09E-14	129
cell cycle	1.50E-18	436	cellular macromolecule catabolic process	6.05E-14	232
rRNA metabolic process	2.00E-18	109	translational termination	1.14E-13	51
alpha-amino acid metabolic process	3.94E-18	103	neurogenesis	1.20E-13	348
cellular amide metabolic process	4.43E-18	283	nuclear-transcribed mRNA catabolic process, nonsense-mediated decay	1.57E-13	58
**Pathways**	**q_FDR**	**Genes**	**Pathways**	**q_FDR**	**Genes**
Metabolism	5.02E-21	439	Metabolism	5.11E-17	434
Metabolism of amino acids and derivatives	3.01E-19	95	Metabolism of amino acids and derivatives	3.34E-11	83
DNA Replication	3.01E-19	65	Extracellular matrix organization	3.34E-11	103
Cholesterol biosynthesis	3.01E-19	159	Steroid Biosynthesis	1.20E-08	19
Synthesis of DNA	6.73E-19	61	cholesterol biosynthetic	2.80E-08	17
S Phase	1.91E-17	69	Switching of origins to a post-replicative state	2.80E-08	39
M/G1 Transition	2.60E-17	54	Orc1 removal from chromatin	2.80E-08	39
DNA Replication Pre-Initiation	2.60E-17	54	Steroid biosynthesis	2.90E-08	18
G1/S Transition	3.13E-16	63	superpathway of cholesterol biosynthesis	2.90E-08	21
Cell Cycle	1.15E-13	170	Cholesterol biosynthesis	2.90E-08	19
Mitotic G1-G1/S phases	8.11E-13	67	M/G1 Transition	3.21E-08	43
Regulation of DNA replication	2.86E-12	45	DNA Replication Pre-Initiation	3.21E-08	43
Assembly of the pre-replicative	2.86E-12	42	Synthesis of DNA	4.18E-08	47
DNA Replication	7.90E-12	31	Removal of licensing factors from origins	4.42E-08	39
M Phase	1.08E-11	92	Cholesterol biosynthesis	4.99E-08	16
Removal of licensing factors from origins	1.23E-11	43	Assembly of the pre-replicative complex	6.16E-08	37

Switching of origins to a post-replicative state	1.82E-11	42	Protein digestion and absorption	1.53E-07	43
Orc1 removal from chromatin	1.82E-11	42	Regulation of DNA replication	1.53E-07	39
Ribosome biogenesis in eukaryotes	9.35E-11	46	Ubiquitin Mediated Degradation of Phosphorylated Cdc25A	1.53E-07	31
Cell Cycle Checkpoints	9.27E-10	57	p53-Independent DNA Damage Response	1.53E-07	31
**Phenotypes**	**q_FDR**	**Genes**	**Phenotypes**	**q_FDR**	**Genes**
embryonic lethality	3.25E-13	445	prenatal lethality	1.29E-08	545
embryonic lethality prior to organogenesis	7.09E-11	200	muscle phenotype	1.18E-06	337
embryonic lethality prior to tooth bud stage	3.00E-10	203	embryonic lethality	1.88E-06	425
embryonic lethality between implantation and placentation	7.97E-10	141	decreased body size	1.97E-06	516
embryo phenotype	7.28E-08	394	lethality during fetal growth through [illegible]	2.07E-06	513
embryonic lethality between implantation and somite formation	8.12E-07	100	decreased total tissue mass	5.79E-05	405
embryonic lethality between implantation and somite formation,	2.68E-06	92	decreased body weight	5.79E-05	405
abnormal muscle fiber morphology	1.11E-05	114	abnormal physical strength	6.71E-05	117
abnormal embryo development	3.18E-05	246	perinatal lethality	6.82E-05	307
abnormal prenatal growth/weight/body size	3.18E-05	254	postnatal lethality	8.04E-05	310
abnormal embryo morphology	5.95E-05	294	premature death	4.41E-04	314
abnormal embryonic growth/weight/body size	5.95E-05	217	abnormal muscle physiology	4.41E-04	224
prenatal lethality, complete penetrance	2.05E-04	104	abnormal muscle morphology	5.03E-04	228
abnormal embryonic tissue physiology	7.75E-04	84	embryogenesis phenotype	5.94E-04	394
abnormal inner cell mass [illegible]	8.23E-04	30	abnormal embryogenesis/ development	5.94E-04	394
abnormal muscle morphology	2.67E-03	214	abnormal cardiovascular system [illegible]	7.45E-04	402
abnormal myocardial fiber morphology	3.90E-03	60	embryonic lethality prior to organogenesis	7.45E-04	168
muscle phenotype	4.69E-03	295	abnormal heart morphology	7.45E-04	291
abnormal cardiovascular system morphology	1.17E-02	381	embryonic lethality prior to tooth bud stage	8.05E-04	169
abnormal prenatal body size	2.14E-02	179	abnormal total tissue mass	1.29E-08	545

Table S3 Top enriched molecular functions, biological processes and cellular components by DAVID using zebrafish genome as background

Molecular Functions	p-value	Gene	%
RNA binding	1.50E-05	27	4.3
translation factor activity, nucleic acid binding	4.60E-04	12	1.9
translation initiation factor activity	1.30E-03	9	1.4
selenium binding	3.00E-03	6	1
tRNA binding	7.90E-03	4	0.6
oxidoreductase activity, acting on single donors with incorporation of molecular oxygen	8.70E-03	6	1

oxidoreductase activity, acting on single donors with incorporation of molecular oxygen	9.90E-03	6	1
iron ion binding	2.10E-02	18	2.9
oxidoreductase activity, acting on sulfur group of donors, disulfide as acceptor	3.70E-02	3	0.5
S-methyltransferase activity	4.60E-02	3	0.5
vascular endothelial growth factor receptor	4.60E-02	3	0.5
adenyl nucleotide binding	5.00E-02	54	8.6
acetyltransferase activity	5.10E-02	5	0.8
purine nucleoside binding	5.30E-02	54	8.6
nucleoside binding	5.70E-02	54	8.6
nucleotide binding	5.80E-02	79	12.5
ATP binding	6.60E-02	51	8.1
adenyl ribonucleotide binding	6.60E-02	51	8.1
purine nucleotide binding	6.80E-02	67	10.6
tRNA (guanine-N7-)-methyltransferase activity	7.70E-02	2	0.3
**Biological Processes**	**p-valule**	**Gene**	**%**
ribonucleoprotein complex biogenesis	5.10E-09	17	2.7
mitosis	3.10E-08	16	2.5
nuclear division	4.10E-08	16	2.5
M phase of mitotic cell cycle	5.40E-08	16	2.5
M phase	7.20E-08	18	2.9
mitotic cell cycle	9.40E-08	17	2.7
cell cycle phase	1.10E-07	18	2.9
organelle fission	1.20E-07	16	2.5
transcription	1.90E-07	55	8.7
ribosome biogenesis	3.50E-07	14	2.2
cell division	1.30E-06	18	2.9
cell cycle process	2.10E-06	18	2.9
cell cycle	2.60E-06	25	4
rRNA processing	4.40E-06	11	1.7
rRNA metabolic process	5.90E-06	11	1.7
protein localization	8.00E-06	36	5.7
protein transport	1.40E-05	34	5.4
establishment of protein localization	1.40E-05	34	5.4
RNA processing	2.30E-05	15	2.4
steroid metabolic process	2.70E-05	26	4.1
**Components**	**p-valule**	**Gene**	**%**
nucleolus	6.50E-14	26	4.1
membrane-enclosed lumen	7.80E-09	44	7
mitochondrion	1.90E-08	48	7.6
organelle lumen	4.70E-08	41	6.5
intracellular organelle lumen	4.70E-08	41	6.5
nuclear lumen	6.30E-07	35	5.5
chromosome, centromeric region	8.80E-07	11	1.7
condensed chromosome kinetochore	3.30E-06	8	1.3
condensed chromosome, centromeric region	3.30E-06	8	1.3
kinetochore	7.50E-06	8	1.3

endosome	9.30E-06	13	2.1
extrinsic to membrane	8.20E-05	18	2.9
condensed chromosome	8.30E-05	8	1.3
spindle	4.30E-04	8	1.3
organelle envelope	6.00E-04	29	4.6
mitochondrial part	6.30E-04	26	4.1
envelope	7.00E-04	29	4.6
mitochondrial membrane	1.00E-03	22	3.5
mitochondrial envelope	1.20E-03	23	3.6
organelle inner membrane	5.90E-03	17	2.7

Table S4 Top 50 statistically enriched canonical pathways and the list of DEGs responsible for their enrichment by IPA

De novo	-log(p-value)	Molecules
Cell Cycle Control of Chromosomal Replication	7.17E+00	MCM5,MCM6,CDC45,ORC2,CDT1,CDC7,RPA1,MCM4,RPA2,MCM3,RPA3,ORC5,ORC4,MCM7
Signaling by Rho Family GTPases	6.05E+00	PIK3R1,ARPC5,GNB5,PIKFYVE,ARHGEF1,CLIP1,STMN1,ROCK2,GNB1,PAK1,RHOG,CFL2,FGFR4,EZR,PIK3CG,MYL4,ACTA1,GNG12,ITGA4,PIK3C2B,MAPK12,KIAA1804,MYL7,CDH2,CDH1,CDH20,IRS1,GNAO1,FNBP1,ARPC1B,RHOT2,GNA14,IQGAP1,JUN,PPP1R12A,CDH3,PIK3C3,RHOU,ARPC3,PI4KA,MYL12A,ITGB1,ITGA2,VIM,CDC42EP3,CDH15,GNAI3,PRKCI,Pak2,CDH17,ARPC4
Unfolded protein response	4.83E+00	PDIA2,CALR,HSPA14,ERN1,INSIG1,HSPA9,CANX,ATF6,OS9,MAP3K5,HSPA4,UBXN4,SREBF1,EDEM1,ERO1B,VCP,AMFR
Axonal Guidance Signaling	4.77E+00	DPYSL2,BMP4,ITSN1,PIK3R1,ARPC5,GNB5,MMP13,NTN1,GNB1,ROCK2,PAK1,SEMA6D,CFL2,SEMA3D,PIK3CG,FGFR4,WNT4,MYL4,GSK3B,CHMP1A,ADAMTS5,GNG12,ITGA4,PIK3C2B,COPS5,TUBB2A,MMP2,HHIP,MYL7,PRKCD,IRS1,GNAO1,FZD6,EPHA2,MME,ADAM17,RND1,ARPC1B,PDGFA,GNA14,PRKAG1,WNT7A,PPP3CB,NGFR,PIK3C3,ARPC3,PRKCE,PSMD14,MMP11,SEMA3B,LNPEP,ACE,BMP1,MYL12A,ITGB1,SEMA3G,PXN,NRP2,WNT9B,CXCR4,ITGA2,EPHA3,PLXND1,BMP5,GNAI3,SEMA3A,FZD8,PRKCI,SEMA4D,FZD4,Pak2,ADAM10,SEMA3C,ADAM9,ARPC4
Mitotic Roles of Polo-Like Kinase	4.70E+00	KIF23,FZR1,ANAPC2,ESPL1,CCNB3,CDC7,PPP2R3B,PLK1,ANAPC1,SLK,PLK4,HSP90AB1,PPP2R3A,CAPN1,HSP90AA1,FBXO5,PPP2R5E,KIF11,CDC25A
RhoGDI Signaling	4.67E+00	ARPC1B,ARPC5,RHOT2,PIKFYVE,GNB5,ARHGEF1,GNA14,ROCK2,GNB1,PAK1,RHOG,PPP1R12A,CFL2,CDH3,EZR,RHOU,ARPC3,MYL4,ARHGAP12,PI4KA,ACTA1,GNG12,MYL12A,ITGA4,ITGB1,ITGA2,CDH15,MYL7,GNAI3,CDH1,CDH2,CDH20,Pak2,GNAO1,CDH17,FNBP1,ARPC4
Integrin Signaling	4.34E+00	ARPC1B,TSPAN7,PIK3R1,RHOT2,ARPC5,PIKFYVE,PPP1CB,ITGA10,TSPAN3,PAK1,RHOG,PPP1R12A,FGFR4,PIK3CG,PIK3C3,RHOU,ITGAV,ARPC3,GSK3B,ITGB4,ACTA1,MYL12A,ITGA4,ITGB1,CAPN5,PIK3C2B,PXN,ITGA2,TNK2,GSN,TTN,MYL7,TSPAN1,IRS1,CAPN1,Pak2,CAPN9,CAPN2,ITGB6,NEDD9,FNBP1,ARPC4
Regulation of Actin-based Motility by Rho	3.90E+00	ITGB1,ARPC1B,RHOT2,ARPC5,ITGA2,PIKFYVE,PPP1CB,GSN,MYL7,PAK1,RHOG,PPP1R12A,Pak2,RHOU,MYL4,ARPC3,FNBP1,ACTA1,ARPC4,PI4KA,ITGA4,MYL12A
Actin Cytoskeleton Signaling	3.80E+00	MYH6,ARPC1B,PDGFA,PIK3R1,ARPC5,PIKFYVE,TRIO,PPP1CB,ARHGEF1,IQGAP1,F2,ROCK2,PAK1,PPP1R12A,CFL2,FLNA,PIK3C3,FGFR4,EZR,PIK3CG,ARPC3,MYL4,ARHGAP24,GNG12,ACTA1,ITGA4,IQGAP3,MYL12A,ITGB1,VAV2,PIK3C2B,PXN,CSK,ITGA2,MYH7,GSN,TTN,MYL7,IRS1,Pak2,SSH3,ARPC4
phagosome maturation	3.79E+00	NOS1,VPS28,PRDX1,PRDX5,TCIRG1,PIKFYVE,DYNLRB2,ATP6AP1,VTI1B,DYNC1I2,PIK3C3,GOSR1,ATP6V1G1,M6PR,ATP6V0E2,CALR,VPS16,VPS41,TUBB2A,RAB7A,CANX,TSG101,ATP6V1F,DYNC1LI2,ATP6V1H,ATP6V0A2,ATP6V0E1
ILK Signaling	3.71E+00	FLNB,MYH6,PIK3R1,RHOT2,PPP2R3B,RHOG,JUN,CFL2,PPP1R12A,FLNA,PIK3CG,FGFR4,PIK3C3,RHOU,MYL4,GSK3B,ITGB4,CTNNB1,ACTA1,ITGB1,PIK3C2B,PXN,CASP3,VIM,MYH7,MAPK12,MYL7,CDH1,FLNC,PPP2R3A,IRS1,LEF1,KRT18,PTGS2,PPP2R5E,ITGB6,FNBP1
Polyamine Regulation in Colon Cancer	3.60E+00	MAX,AZIN1,PSMF1,MXD1,SAT1,SAT2,CTNNB1,PSME3,ODC1
Protein Ubiquitination Pathway	3.60E+00	FZR1,ANAPC2,UBE2A,HSPA14,PSMD9,USP2,ANAPC1,PSMC5,HSPA4,PSMD10,HSP90AB1,PSMC6,HSPE1,USP16,UCHL5,SUGT1,PSMA3,PSMD14,DNAJB1,PSMC2,AMFR,PSMB4,PSMA6,DNAJB12,UBE4B,USP38,PSMD13,HSPA9,PSMD6,PSMA1,THOP1,PSMB6,UBE3A,DNAJB14,PSMD8,SKP2,DNAJC11,PSMD2,PSMD12,PSMA4,HSP90AA1,PSMD1,PSMD4,DNAJC7,UBE2E1
Gα12/13 Signaling	3.56E+00	PIK3R1,ARHGEF1,MAP3K5,F2,ROCK2,IKBKB,JUN,NFKBIA,CDH3,PIK3C3,PIK3CG,FGFR4,MYL4,CTNNB1,MYL12A,VAV2,PIK3C2B,PXN,CDH15,MAPK12,MYL7,CDH2,CDH1,CDH20,IRS1,CDH17,MEF2C
Hepatic Fibrosis / Hepatic Stellate Cell Activation	3.55E+00	MYH6,PDGFA,MMP13,COL8A1,COL6A6,COL5A1,COL1A2,COL6A1,NGFR,HGF,MYL4,ECE1,COL11A2,STAT1,COL18A1,COL5A2,COL4A1,COL9A1,COL6A2,COL2A1,MYH7,MMP2,IGFBP5,IL1R1,MYL7,COL5A3,COL21A1,COL23A1,COL24A1,EDNRA,COL11A1,KDR,COL9A2,A2M,COL7A1
Mechanisms of Viral Exit from Host Cells	3.25E+00	SH3GL1,PRKCI,CHMP2B,VPS28,PRKCD,CHMP4B,PRKCE,PDCD6IP,TSG101,LMNB1,CHMP3,ACTA1

Assembly of RNA Polymerase I Complex	3.02E+00	POLR1D,POLR1C,POLR1A,POLR1B,TAF1C
Remodeling of Epithelial Adherens Junctions	2.97E+00	ARPC1B,TUBB2A,ARPC5,RAB7A,CTNNA1,IQGAP1,CLIP1,CDH1,HGF,ARPC3,CTNNB1,DNM1L,HGS,ARPC4,ACTA1,CTNND1
Molecular Mechanisms of Cancer	2.90E+00	BMP4,SUV39H1,PIK3R1,ARHGEF1,RBL1,PAK1,CAMK2A,RHOG,FGFR4,PIK3CG,WNT4,GSK3B,SMAD1,CDC25A,ITGA4,PIK3C2B,CCNE2,CASP3,MAPK12,RALBP1,CDH1,MAX,IRS1,PRKCD,E2F1,GNAO1,FZD6,APH1B,LEF1,FNBP1,CAMK2G,PA2G4,RHOT2,CTNNA1,GNA14,MAP3K5,E2F3,PRKAG1,NFKBIA,JUN,WNT7A,PIK3C3,RHOU,PRKCE,CTNNB1,CAMK2B,BMP1,ITGB1,WNT9B,ITGA2,BMP5,GNAI3,FZD8,PRKCI,FZD4,NF1,Pak2,CTNND1
Tec Kinase Signaling	2.89E+00	PIK3R1,RHOT2,GNB5,GNA14,GNB1,PAK1,RHOG,PIK3C3,PIK3CG,FGFR4,RHOU,PRKCE,STAT1,STAT5B,GNG12,ACTA1,ITGA4,VAV2,ITGB1,PIK3C2B,ITGA2,STAT3,MAPK12,FRK,GNAI3,PRKCI,PRKCD,IRS1,Pak2,GNAO1,FNBP1
HGF Signaling	2.78E+00	ETS1,ITGB1,PIK3C2B,PXN,PIK3R1,ITGA2,MAP3K5,STAT3,MAPK12,ELF4,ELF2,PAK1,JUN,PRKCI,PIK3CG,FGFR4,IRS1,PRKCD,PIK3C3,HGF,PRKCE,PTGS2,ITGA4
Amyloid Processing	2.77E+00	CSNK1E,CAPN5,CSNK2A1,CAPN1,CSNK1D,PRKCE,APH1B,CAPN9,CAPN2,BACE2,GSK3B,MAPK12,PRKAG1
Semaphorin Signaling in Neurons	2.69E+00	ROCK2,ITGB1,DPYSL2,SEMA3A,PAK1,RHOG,SEMA4D,RND1,CFL2,Pak2,RHOT2,RHOU,FNBP1
Rac Signaling	2.68E+00	ITGB1,PIK3C2B,RPS6KB1,ARPC1B,PIK3R1,ITGA2,ARPC5,PIKFYVE,IQGAP1,PAK1,PRKCI,JUN,CFL2,PIK3CG,FGFR4,IRS1,PIK3C3,Pak2,ARPC3,ARPC4,PI4KA,ITGA4,IQGAP3
CXCR4 Signaling	2.59E+00	PIK3R1,RHOT2,GNB5,GNA14,ROCK2,GNB1,ELMO3,PAK1,RHOG,JUN,PIK3CG,FGFR4,PIK3C3,RHOU,PRKCE,MYL4,GNG12,MYL12A,PIK3C2B,PXN,CXCR4,MAPK12,MYL7,GNAI3,PRKCI,PRKCD,IRS1,Pak2,GNAO1,FNBP1
NF-κB Activation by Viruses	2.59E+00	ITGB1,PIK3C2B,PIK3R1,ITGA2,TNFRSF14,IKBKB,NFKBIA,RIPK1,PRKCI,PIK3CG,IRS1,PIK3C3,FGFR4,PRKCD,ITGAV,PRKCE,IKBKAP,ITGA4
Small Cell Lung Cancer Signaling	2.59E+00	NOS1,PIK3C2B,CCNE2,FHIT,PA2G4,SUV39H1,PIK3R1,SKP2,IKBKB,NFKBIA,MAX,PIK3C3,PIK3CG,FGFR4,IRS1,TRAF4,E2F1,PTGS2
HIPPO signaling	2.59E+00	DLG1,TJP2,CSNK1D,PPP1CB,PPP2R3B,LATS2,STK3,DLG3,SKP2,CSNK1E,Ppp1cc,PPP1R10,PPP1R12A,PPP2R3A,PPP2R5E,PATJ,SMAD1,RASSF1
Virus Entry via Endocytic Pathways	2.57E+00	ITGB1,PIK3C2B,FLNB,ITSN1,PIK3R1,ITGA2,AP2A2,PRKCI,FLNC,FLNA,PIK3CG,FGFR4,IRS1,PRKCD,PIK3C3,CLTA,PRKCE,ITGB4,ITGB6,ACTA1,ITGA4
HER-2 Signaling in Breast Cancer	2.53E+00	ITGB1,TSC1,PIK3C2B,CCNE2,PIK3R1,MMP2,ERBB3,MAP3K5,PRKCI,PIK3C3,PIK3CG,PRKCD,IRS1,FGFR4,PRKCE,ITGB4,GSK3B,ITGB6
Role of p14/p19ARF in Tumor Suppression	2.50E+00	NPM1,PIK3C2B,IRS1,FGFR4,PIK3CG,PIK3R1,PIK3C3,E2F1,SENP3,SF3A1,POLR3D
Ephrin A Signaling	2.49E+00	ROCK2,VAV2,PIK3C2B,PAK1,CFL2,PIK3C3,PIK3CG,NGFR,FGFR4,IRS1,PIK3R1,ADAM10,EPHA3,EPHA2
Germ Cell-Sertoli Cell Junction Signaling	2.48E+00	PIK3R1,RHOT2,CTNNA1,MAP3K5,IQGAP1,PAK1,RHOG,CFL2,SORBS1,PIK3C3,FGFR4,PIK3CG,RHOU,CTNNB1,ACTA1,ITGB1,PIK3C2B,PXN,TUBB2A,ITGA2,MAPK12,GSN,CDH2,CDH1,IRS1,Pak2,A2M,FNBP1,CTNND1,NECTIN2
PAK Signaling	2.47E+00	ITGB1,PIK3C2B,PXN,CASP3,PDGFA,PIK3R1,ITGA2,EPHA3,MAPK12,MYL7,PAK1,CFL2,PIK3CG,FGFR4,IRS1,PIK3C3,Pak2,MYL4,ITGA4,MYL12A
Role of Osteoblasts, Osteoclasts and Chondrocytes in Rheumatoid Arthritis	2.46E+00	ADAM17,BMP4,MMP3,PIK3R1,MMP14,MMP13,SP7,MAP3K5,IKBKB,WNT7A,JUN,NFKBIA,PPP3CB,PIK3C3,FGFR4,NGFR,PIK3CG,WNT4,GSK3B,CTNNB1,ADAMTS5,SMAD1,BMP1,ITGB1,PIK3C2B,CTSK,WNT9B,ITGA2,IL1R1,MAPK12,GSN,BMP5,FZD8,FZD4,IRS1,FZD6,LEF1,SFRP1
Actin Nucleation by ARP-WASP Complex	2.46E+00	ROCK2,ITGB1,RHOG,PPP1R12A,ARPC1B,ITGA2,ARPC5,RHOT2,RHOU,ARPC3,ARPC4,FNBP1,ITGA4
Ephrin Receptor Signaling	2.44E+00	ITSN1,ARPC1B,PDGFA,ARPC5,GNB5,GNA14,GNB1,ROCK2,PAK1,CFL2,SORBS1,PIK3CG,ARPC3,GNG12,ITGA4,ITGB1,GRIN1,PXN,CXCR4,ITGA2,EPHA3,STAT3,GNAI3,Pak2,GNAO1,ADAM10,DOK1,MAP4K4,EPHA2,ARPC4
Huntington's Disease Signaling	2.43E+00	POLR2F,HSPA14,SGK1,PIK3R1,GNB5,GNA14,VTI1B,AP2A2,GNB1,HSPA4,DYNC1I2,POLR2A,JUN,PIK3C3,FGFR4,PIK3CG,PRKCE,NCOR1,DNAJB1,GOSR1,GNG12,CAPN5,SDHA,PIK3C2B,CASP3,PSMF1,GLS,HSPA9,HIP1,PSME3,PRKCI,CLTA,PRKCD,IRS1,CAPN1,PENK,CAPN9,CAPN2,DNM1L
NRF2-mediated Oxidative Stress Response	2.41E+00	PRDX1,PIK3R1,MAP3K5,SOD3,GSTT1,JUN,PIK3C3,ABCC1,FGFR4,PIK3CG,VCP,UBE2K,PRKCE,DNAJA3,GSK3B,DNAJB1,CBR1,ACTA1,PIK3C2B,DNAJB12,SOD1,DNAJB14,GSTO1,TXNRD1,DNAJC11,PRKCI,PRKCD,IRS1,CCT7,ENC1,DNAJC7,MGST3
IL-8 Signaling	2.40E+00	PIK3R1,RHOT2,GNB5,IQGAP1,EIF4EBP1,ROCK2,GNB1,IKBKB,RHOG,JUN,PIK3CG,FGFR4,PIK3C3,RHOU,ITGAV,PRKCE,GNG12,PIK3C2B,RPS6KB1,PLD3,HBEGF,MMP2,MAPK12,MYL7,GNAI3,CDH1,PRKCI,PRKCD,IRS1,PTGS2,KDR,MAP4K4,FNBP1
Type II Diabetes Mellitus Signaling	2.40E+00	PIK3C2B,PRKAB2,PRKAB1,PKLR,PIK3R1,PKM,ADIPOQ,SMPD1,MAP3K5,MAPK12,PRKAG1,KCNJ11,IKBKB,PRKCI,NFKBIA,SMPD4,PRKCD,IRS1,PIK3C3,PIK3CG,FGFR4,NGFR,ACSL5,PRKCE,SLC27A1,ACSL1
ERK/MAPK Signaling	2.34E+00	PIK3R1,PPP2R3B,PPP1CB,PRKAG1,KSR1,EIF4EBP1,ELF4,PAK1,PPP1R10,PPP1R12A,PIK3CG,FGFR4,PIK3C3,PRKCE,STAT1,PLA2G12B,ITGA4,ITGB1,ETS1,PIK3C2B,PXN,ITGA2,PLA2G3,MAPKAPK5,STAT3,ELF2,PRKCI,DUSP1,PPP2R3A,PRKCD,IRS1,Pak2,PPP2R5E
Fcγ Receptor-mediated Phagocytosis in Macrophages and Monocytes	2.32E+00	VAV2,RPS6KB1,PXN,PLD3,ARPC1B,PIK3R1,ARPC5,MYO5A,PAK1,PRKCI,RAB11B,PIK3CG,EZR,PRKCD,RAB11A,PRKCE,ARPC3,ACTA1,ARPC4
nNOS Signaling in Neurons	2.29E+00	CAPN5,NOS1,GRIN1,PRKCI,CAMK2A,PPP3CB,PRKCD,CAPN1,PRKCE,CAPN9,CAPN2,PFKM
Tight Junction Signaling	2.25E+00	MYH6,CTNNA1,PPP2R3B,VTI1B,PRKAG1,CPSF4,OCLN,JUN,CLDN4,NGFR,MYL4,GOSR1,CTNNB1,ACTA1,TJP2,RAB13,MYH7,MYL7,F11R,PRKCI,CLDN23,CLDN8,PPP2R3A,CPSF3,PPP2R5E,PATJ,CLDN3,NECTIN2
AMPK Signaling	2.24E+00	TSC1,PRKAB1,PRKAB2,PIK3R1,CPT1B,CHRNB1,ADIPOQ,PPP2R3B,PFKL,PRKAG1,ELAVL1,EIF4EBP1,CCNA2,GYS1,FASN,FGFR4,PIK3CG,PIK3C3,RPTOR,HNF4A,RPS6KB1,PIK3C2B,ACACB,MAPK12,SMARCD3,PFKM,AK6,PPM1B,PPP2R3A,IRS1,ACACA,PPP2R5E,HMGCR
Regulation of eIF4 and p70S6K Signaling	2.19E+00	PABPC1,ITGB1,PIK3C2B,RPS6KB1,EIF3C,EIF4EBP2,PIK3R1,ITGA2,PPP2R3B,MAPK12,EIF2S1,EIF4G1,EIF2S2,FAU,EIF4EBP1,EIF4G2,EIF3B,PPP2R3A,IRS1,PIK3CG,FGFR4,PIK3C3,RPS27L,PPP2R5E,AGO1,

		EIF1AX,ITGA4
Ovarian Cancer Signaling	2.19E+00	PIK3C2B,RPS6KB1,PA2G4,WNT9B,SUV39H1,PIK3R1,PTGS1,MMP2,PRKAG1,FZD8,FZD4,WNT7A,MSH2,PIK3C3,PIK3CG,FGFR4,IRS1,E2F1,FZD6,WNT4,EDNRA,LEF1,PTGS2,GSK3B,CTNNB1

OnRamp	-log(p-value)	Molecules
EIF2 Signaling	2.13E+01	RPL11,RPS27,PIK3R1,SOS2,RPL39,PDPK1,RPL35A,RPS11,FGFR3,RPL13,RPS20,EIF3B,RPS13,EIF3D,PIK3CG,RPL23A,INS,EIF5,RPL21,PPP1CA,AGO1,ATM,RPL32,FGFR1,RPS8,RPL7L1,FGFR2,RPL12,EIF2S3,RPL37A,TLR9,RPL9,EIF3M,RPS6,RPL8,IRS1,RPL10,RPL6,RPS15A,INSR,RPL13A,RPSA,EIF3K,RPL36A,RPS18,PPP1CB,RPL7A,EIF4G1,EIF4E,RPL7,RPS4X,PPP1CC,RPL18A,KL,UBA52,PIK3C3,RPS9,EIF3A,RPS3,RPS17,RPL18,RPS19,EIF3H,RPL34,RPL17,PIK3C2G,RPL30,RPS10,RPL23,RPS29,RPL27,EIF3F,RPS15,RPS16,RPS26,RPS27A,RPL5,PIK3CB,RPL37,EIF3L,EIF1AX,RPS14
Superpathway of Cholesterol Biosynthesis	1.23E+01	MVD,SQLE,NSDHL,ACAT2,IDI1,MVK,HSD17B7,MSMO1,TM7SF2,SC5D,LBR,FDPS,FDFT1,EBP,DHCR7,DHCR24,ACAT1,LSS,HMGCR,HMGCS1,CYP51A1
Axonal Guidance Signaling	1.12E+01	DPYSL2,PAPPA2,ADAMTS8,ECEL1,BMP4,ITSN1,PIK3R1,SOS2,ARPC5,ADAM8,NTN1,GNB1,ECE2,PIK3CG,ABLIM3,PTCH2,ATM,EFNA2,COPS5,FGFR2,MMP2,TLR9,ADAMTS6,ARPC1A,IRS1,RTN4,EPHA2,GNAL,NRP1,MME,UNC5A,ARPC1B,NOTUM,ABLIM1,EIF4E,GNG7,WNT7A,TUBA8,KL,PIK3C3,DCC,ADAM19,ARPC3,PSMD14,LNPEP,BMP1,MYL12A,SEMA3G,ADAMTS13,NRP2,MYLPF,PIK3C2G,SEMA3A,GLIS2,MAG,ADAM10,BMP7,SEMA3C,BMP6,ARPC4,NTN3,PRKCB,WNT3,RACK1,CXCL12,GNB5,MYL6B,ADAM11,TUBB,FGFR3,SEMA6D,GNB3,WNT7B,ADAM23,WNT4,ADAMTS5,FGFR1,PPP3CC,HHIP,RAC3,MYL7,CDC42,RHOA,PRKCD,FZD6,GNAT2,PRKCH,RND1,SLIT1,PDGFA,BMP3,SEMA4C,WNT2,NFATC1,CDK5,GLIS1,MKNK1,MMP11,SEMA3B,SHANK2,SEMA3F,ACE,VASP,PRKCA,PXN,WNT9B,CXCR4,TUBG1,PRKAR2A,GNAI1,PLXND1,GNAI2,PRKCI,WNT10A,LINGO1,PIK3CB,GLI1
Cholesterol Biosynthesis I	1.11E+01	SQLE,FDFT1,EBP,NSDHL,DHCR7,DHCR24,HSD17B7,MSMO1,LSS,TM7SF2,SC5D,CYP51A1,LBR
Cholesterol Biosynthesis II (via 24,25-dihydrolanosterol)	1.11E+01	SQLE,FDFT1,EBP,NSDHL,DHCR7,DHCR24,HSD17B7,MSMO1,LSS,TM7SF2,SC5D,CYP51A1,LBR
Cholesterol Biosynthesis III (via Desmosterol)	1.11E+01	SQLE,FDFT1,EBP,NSDHL,DHCR7,DHCR24,HSD17B7,MSMO1,LSS,TM7SF2,SC5D,CYP51A1,LBR
Type II Diabetes Mellitus Signaling	1.01E+01	MAP2K4,RELA,SLC27A2,PRKAB2,PKLR,PIK3R1,PKM,PDPK1,SMPD1,MAP3K5,ADIPOR1,SLC27A4,FGFR3,IKBKB,NFKBIA,ACSBG1,KL,PIK3C3,PIK3CG,ACSL5,INS,ABCC8,PRKAA2,CHUK,ATM,PRKCA,PPARG,FGFR1,ACSL6,PIK3C2G,FGFR2,TLR9,TRAF2,PRKCI,PRKCD,IRS1,GCK,MAPK10,PIK3CB,SLC27A1,PRKCH,SLC27A3,INSR,ADIPOR2,SOCS5,ACSL1,PRKCB
Regulation of eIF4 and p70S6K Signaling	1.01E+01	PPP2CA,RPS27,PIK3R1,SOS2,PDPK1,PPP2R3B,RPS11,FGFR3,EIF3B,RPS20,EIF3D,RPS13,PIK3CG,AGO1,ATM,RPS6KB1,FGFR1,RPS8,FGFR2,EIF2S3,TLR9,EIF3M,RPS6,IRS1,RPS15A,EIF3K,RPSA,EIF4EBP2,RPS18,EIF4G1,EIF4E,RPS4X,KL,PIK3C3,MKNK1,RPS9,EIF3A,RPS3,RPS17,RPS19,EIF3H,PIK3C2G,RPS10,RPS29,RPS15,EIF3F,RPS16,RPS26,RPS27A,PIK3CB,PPP2R5E,EIF1AX,EIF3L,RPS14
Protein Ubiquitination Pathway	9.10E+00	FZR1,UBE2A,HSPA5,PSMC5,UBE2D4,PAN2,USP8,USP10,UCHL5,USP40,PSMA2,ANAPC11,PSMC2,PSMA6,PSMB5,UBE4B,PSMC4,BIRC6,PSMD6,DNAJC2,PSMD3,THOP1,HSPA8,PSMD11,PSMB7,UBE2D2,UBE2H,RBX1,PSMD12,PSMA5,PSMA4,PSMB1,HSP90AA1,PSMD1,UBE2I,ANAPC2,PSMB3,USP24,USP14,CDC20,PSMD7,HSPB8,DNAJC10,CDC23,USP39,ANAPC1,HSPA1L,SMURF1,HSPA12B,USP13,HSP90AB1,PSMC6,USP16,USP47,PSMD14,PSMA3,DNAJB1,AMFR,PSMB4,PSMD13,MDM2,PSMA1,HSPD1,HSPA2,USP33,UBE2J1,USP4,PSMD2,DNAJB11,CDC34,UBA1,USP34,PSMC3
mTOR Signaling	8.97E+00	PRKAB2,PPP2CA,RPS27,PIK3R1,PDPK1,PPP2R3B,RPS11,FGFR3,RPS20,EIF3B,EIF3D,RPS13,PIK3CG,INS,ATM,RPS6KB1,FGFR1,RPS8,FGFR2,TLR9,EIF3M,RPS6,RHOQ,RND3,PRKCD,IRS1,RHOA,PRKCH,RPS15A,INSR,RPSA,EIF3K,RPS18,EIF4G1,RPS4X,EIF4E,KL,PIK3C3,RPS9,PRKAA2,EIF3A,RPS17,RPS3,PRKCA,RPS19,EIF3H,RPS10,PIK3C2G,RPS29,DGKZ,EIF3F,RPS15,RPS16,PRKCI,RPS26,RPS27A,PIK3CB,PPP2R5E,EIF3L,PRKCB,RPS14
Amyotrophic Lateral Sclerosis Signaling	8.31E+00	NOS1,GRIN2A,CACNA1S,PIK3R1,GRIA1,GRIN2D,GRID2,GRIA4,FGFR3,KL,PIK3C3,PIK3CG,ATM,GRIN1,GRIN2B,CAPN6,CACNA1D,CASP3,FGFR1,PIK3C2G,APAF1,FGFR2,CACNA1C,BAX,SOD1,TLR9,ALS2,CACNA1A,CCS,CAPNS1,GRIK4,IRS1,CAPN1,CAT,GLUL,PIK3CB,CAPN9,CAPN2,CAPN3,GRIA3
Protein Kinase A Signaling	7.27E+00	ENPP6,MYH10,PDE6G,BAD,RACK1,GNB5,LIPE,MYL6B,NTN1,GNB1,GYS1,CAMK2A,GNB3,RHO,RYR3,DUSP7,ANAPC11,PPP1CA,APEX1,PTCH2,GRK7,CDC25A,SMPDL3A,PTPRE,YWHAB,PDE10A,ITPR2,PDE6C,PTPN3,ITPR1,PPP3CC,PDE4B,CNGA1,CNGA3,GYS2,MYL7,PDE8A,PYGM,H3F3A/H3F3B,RHOA,PRKCD,PTPRB,ITPR3,EYA3,PTPRS,PRKCH,CAMK2G,ANAPC2,RELA,PTPN9,NOTUM,PPP1CB,CDC23,ANAPC1,GNG7,NFATC1,MPPE1,PPP1CC,NFKBIA,PPP1R10,FLNA,CREB1,DCC,SMAD4,PTPRZ1,CNGB3,CHUK,CTNNB1,PTPRT,VASP,PRKCA,CAMK2B,MYL12A,PXN,PTPRK,MYLPF,RYR2,ADCY3,GNAI1,PRKAR2A,PYGB,TCF7L1,GNAI2,PRKCI,HIST1H1E,FLNC,CDC14B,ADCY1,KDELR3,IHH,PDE5A,PTGS2,PTPRR,PRKCB
Huntington's Disease Signaling	7.09E+00	MAP2K4,POLR2F,SGK1,PIK3R1,SOS2,RACK1,GNB5,PDPK1,HSPA5,FGFR3,GNB1,CTSD,GNB3,PIK3CG,CASQ1,TCERG1,DLG4,POLR2L,ATM,CAPN6,CASP3,FGFR1,FGFR2,DNM3,ITPR1,TLR9,RPH3A,HSPA8,CACNA1B,CAPN1,PRKCD,IRS1,PSME4,PRKCH,CAPN2,CAPN3,SDHB,HSPA1L,GNG7,TGM2,DYNC1I2,ARFIP2,CDK5,KL,PIK3C3,CREB1,DNAJB1,PRKCA,NEUROD1,SDHA,GRIN2B,HDAC1,APAF1,PIK3C2G,BAX,HIP1,HSPA2,PSMA8,PRKCI,CAPNS1,PIK3CB,CAPN9,NCOR2,DNM1L,PRKCB
Glycolysis I	6.91E+00	PKLR,ENO3,PKM,PGAM2,PFKL,PFKP,BPGM,PFKM,GPI,ALDOB,PGAM1,FBP1,ALDOA,GAPDH,GAPDHS
Hepatic Fibrosis / Hepatic Stellate Cell Activation	6.57E+00	MYH10,KLF6,COL8A1,MYL6B,MYH7B,COL11A2,COL27A1,TIMP2,COL9A1,FGFR1,COL2A1,BAMBI,FGFR2,MMP2,IGFBP5,MYL7,COL21A1,COL23A1,IGFBP3,IL1RAPL1,COL7A1,RELA,COL8A2,MYH6,COL4A5,FN1,COL4A6,PDGFA,COL10A1,MYH11,COL6A6,COL5A1,COL1A2,COL6A1,HGF,PDGFRA,COL22A1,SMAD4,ECE1,STAT1,MYH1,COL5A2,EDNRB,COL6A2,COL12A1,SMAD7,BAX,COL1A1,COL5A3,EDNRA,COL11A1,COL9A2
Thrombin Signaling	6.55E+00	PIK3R1,RACK1,GNB5,PDPK1,MYL6B,GATA2,F2,FGFR3,GNB1,IKBKB,CAMK2A,GNB3,PIK3CG,ATM,RPS6KB1,ITPR2,FGFR1,FGFR2,ITPR1,TLR9,MYL7,RHOQ,RND3,ARHGEF16,RHOA,PRKCD,IRS1,ITPR3,GNAT2,PRKCH,GNAL,CAMK2G,RELA,GATA1,NOTUM,PPP1CB,GNG7,KL,PIK3C3,CREB1,CAMK1G,GATA6,MYL12A,PRKCA,CAMK2B,MYLPF,ADCY3,PIK3C2G,GNAI1,GNAI2,PRKCI,ADCY1,PIK3CB,GATA3,GATA4,PRKCB
AMPK Signaling	6.48E+00	ADRA2B,PRKAB2,PPP2CA,PIK3R1,CPT1B,CHRNB1,PPP2R3B,LIPE,PDPK1,ARID2,FGFR3,AK1,GYS1,PI

		K3CG,INS,HNF4A,ATM,PFKFB3,RPS6KB1,ACACB,SLC2A1,EEF2,FGFR1,AK3,FGFR2,TLR9,SMARCD3,GYS2,PFKM,IRS1,CPT2,ACACA,INSR,PBRM1,ARID1A,PFKL,KL,PIK3C3,CREB1,PRKAA2,EEF2K,CKM,STRADA,PRKAR2A,PIK3C2G,PFKP,FOXO1,PPM1B,PIK3CB,CHRNE,PPP2R5E,SMARCC1,HMGCR
nNOS Signaling in Neurons	6.46E+00	NOS1,GRIN1,GRIN2B,CAPN6,GRIN2A,GRIN2D,DLG2,PPP3CC,PFKM,CAMK2A,CAPNS1,PRKCI,PRKCD,CAPN1,DLG4,CAPN9,CAPN2,PRKCH,CAPN3,PRKCA,PRKCB
fMLP Signaling in Neutrophils	6.15E+00	RELA,ARPC1B,PIK3R1,RACK1,ARPC5,GNB5,GNG7,NFATC1,FGFR3,GNB1,GNB3,NFKBIA,KL,PIK3CG,PIK3C3,CYBB,ARPC3,ATM,PRKCA,ITPR2,FGFR1,PIK3C2G,GNAI1,FGFR2,PPP3CC,ITPR1,TLR9,GNAI2,PRKCI,ARPC1A,CDC42,PRKCD,IRS1,ITPR3,PIK3CB,PRKCH,ARPC4,PRKCB
Role of NANOG in Mammalian Embryonic Stem Cell Pluripotency	6.05E+00	IL6ST,JAK1,BMP4,WNT3,AXIN1,PIK3R1,BMP3,SOS2,WNT2,FGFR3,SOX2,WNT7A,KL,WNT7B,PIK3CG,PIK3C3,GATA6,WNT4,SMAD4,CTNNB1,ATM,BMP1,SMAD9,WNT9B,FGFR1,PIK3C2G,FGFR2,TCF7L1,STAT3,TLR9,WNT10A,IRS1,FZD6,PIK3CB,BMP7,BMP6,GATA4,SALL4
Neuropathic Pain Signaling In Dorsal Horn Neurons	5.92E+00	GRIN2A,PIK3R1,GRIA1,GRIN2D,NOTUM,GRIA4,FGFR3,KCNN1,CAMK2A,KL,PIK3C3,PIK3CG,CREB1,CAMK1G,GRM6,PRKCA,CAMK2B,ATM,GRIN1,GRIN2B,ITPR2,FGFR1,PIK3C2G,PRKAR2A,FGFR2,ITPR1,TLR9,PRKCI,PRKCD,IRS1,ITPR3,PIK3CB,PRKCH,GRIA3,CAMK2G,PRKCB
CREB Signaling in Neurons	5.72E+00	GRIN2A,POLR2F,PIK3R1,RACK1,SOS2,GNB5,GRIA4,FGFR3,GNB1,GNB3,CAMK2A,PIK3CG,GRM6,POLR2L,ATM,ITPR2,FGFR1,FGFR2,ITPR1,TLR9,IRS1,PRKCD,ITPR3,GNAT2,PRKCH,GNAL,CAMK2G,GRIA1,GRIN2D,GRID2,NOTUM,GNG7,KL,PIK3C3,CREB1,CAMK2B,PRKCA,GRIN2B,GRIN1,ADCY3,GNAI1,PRKAR2A,PIK3C2G,GNAI2,PRKCI,GRIK4,ADCY1,PIK3CB,PRKCB,GRIA3
Leukocyte Extravasation Signaling	5.68E+00	MAP2K4,MMP20,MMP16,PIK3R1,CXCL12,CLDN7,FGFR3,PIK3CG,CYBB,ARHGAP12,ATM,TIMP2,FGFR1,ITGA6,FGFR2,MMP2,RAPGEF3,TLR9,MMP23B,CLDN23,CLDN8,JAM3,CDC42,RHOA,PRKCD,IRS1,MAPK10,PRKCH,CTTN,CLDN15,MMP14,MMP15,RAPGEF4,KL,PIK3C3,MMP11,CTNNB1,VASP,ACTN1,PRKCA,VAV2,CLDN10,PXN,CXCR4,ACTN2,GNAI1,PIK3C2G,GNAI2,CLDN5,PRKCI,ARHGAP9,RASGRP1,PIK3CB,PRKCB,MSN
Maturity Onset Diabetes of Young (MODY) Signaling	5.32E+00	NEUROD1,CACNA1S,CACNA1D,PKLR,ALDOB,GCK,INS,CACNA1C,GAPDH,INSR,HNF4A,CACNA1A
eNOS Signaling	5.26E+00	PRKAB2,PIK3R1,CHRNB1,SLC7A1,PDPK1,HSPA5,HSPA1L,FGFR3,NOSIP,LPAR2,HSP90AB1,KL,PIK3CG,PIK3C3,PRKAA2,CNGB3,PRKCA,ATM,CASP3,ITPR2,FGFR1,ADCY3,PIK3C2G,PRKAR2A,FGFR2,AQP1,ITPR1,TLR9,HSPA2,CNGA1,CNGA3,HSPA8,AQP9,PRKCI,PRKCD,IRS1,ADCY1,ITPR3,HSP90AA1,CHRNE,PIK3CB,PRKCH,PRKCB
CXCR4 Signaling	5.26E+00	MAP2K4,PIK3R1,RACK1,CXCL12,GNB5,MYL6B,GNG7,FGFR3,GNB1,ELMO3,GNB3,KL,PIK3CG,PIK3C3,MYL12A,PRKCA,ATM,PXN,ITPR2,CXCR4,EGR1,FGFR1,MYLPF,ADCY3,GNAI1,PIK3C2G,FGFR2,ITPR1,TLR9,MYL7,GNAI2,PRKCI,RHOQ,RND3,PRKCD,IRS1,RHOA,ADCY1,ITPR3,MAPK10,GNAT2,PIK3CB,PRKCH,GNAL,PRKCB
Molecular Mechanisms of Cancer	5.25E+00	MAP2K4,BMP4,JAK1,WNT3,BAD,AXIN1,PIK3R1,SOS2,MYC,FGFR3,RASGRF1,CAMK2A,WNT7B,PIK3CG,WNT4,HIPK2,PTCH2,ATM,CDC25A,CASP3,SMAD9,FGFR1,FGFR2,RAPGEF3,TLR9,RAC3,RALBP1,RASGRF2,CDH1,RHOQ,RND3,CDC42,ARHGEF16,RHOA,PRKCD,IRS1,FZD6,MAPK10,GNAT2,PRKCH,CDK2,GNAL,CAMK2G,RELA,PA2G4,BMP3,PSEN2,MAP3K5,WNT2,SYNGAP1,WNT7A,NFKBIA,CDK5,KL,PIK3C3,SMAD4,CTNNB1,BMP1,CAMK2B,PRKCA,PRKDC,WNT9B,ADCY3,PRKAR2A,GNAI1,SMAD7,APAF1,PIK3C2G,MDM2,BAX,GNAI2,PRKCI,WNT10A,FOXO1,NF1,RASGRP1,ADCY1,IHH,PIK3CB,ATR,BMP7,BMP6,GLI1,PRKCB
GABA Receptor Signaling	5.20E+00	AP2A1,AP2M1,ABAT,GABRA4,ADCY3,GABBR1,GPHN,ALDH9A1,GABRP,GABRB2,SLC6A11,GABRG3,KCNN1,GABRR2,GAD2,GABRG2,GABRB3,GAD1,ADCY1,GABRA6,SLC6A1,GABARAP,GABRD,GABRA1
Clathrin-mediated Endocytosis Signaling	5.16E+00	APOB,PIK3R1,ARPC5,SH3GL2,F2,FGFR3,SNX9,PIK3CG,INS,AMPH,ITGB4,SH3KBP1,ATM,MYO6,FGFR1,FGFR2,DNM3,PPP3CC,TLR9,HSPA8,ARPC1A,PIP5K1C,CDC42,IRS1,CTTN,RBP4,APOD,AP2A1,APOA4,ARPC1B,PDGFA,FGF13,KL,PIK3C3,ARPC3,HGS,FGF7,AP2M1,RAB7A,PIK3C2G,MDM2,HIP1,TSG101,LDLR,SYNJ1,TF,PIK3CB,DNM1L,CLU,ARPC4,HIP1R
Zymosterol Biosynthesis	5.12E+00	NSDHL,HSD17B7,MSMO1,TM7SF2,CYP51A1,LBR
Nitric Oxide Signaling in the Cardiovascular System	5.11E+00	CACNA1S,PIK3R1,SLC7A1,ARG2,ATP2A2,FGFR3,HSP90AB1,KL,PIK3C3,PIK3CG,PRKCA,ATM,CACNA1D,GUCY2D,ITPR2,FGFR1,RYR2,PIK3C2G,PRKAR2A,ATP2A3,CACNA1C,FGFR2,ITPR1,TLR9,CACNA1A,PRKCI,PRKCD,IRS1,ITPR3,HSP90AA1,PDE5A,PIK3CB,PRKCH,PRKCB
Breast Cancer Regulation by Stathmin1	5.10E+00	PPP2CA,PIK3R1,RACK1,SOS2,GNB5,PPP2R3B,TUBB,FGFR3,GNB1,GNB3,CAMK2A,PIK3CG,PPP1CA,ATM,ITPR2,FGFR1,FGFR2,ITPR1,TLR9,CDC42,RHOA,IRS1,PRKCD,ARHGEF16,ITPR3,PRKCH,CDK2,CAMK2G,PPP1CB,GNG7,PPP1CC,PPP1R10,TUBA8,KL,PIK3C3,RB1CC1,CAMK1G,CAMK2B,PRKCA,TUBG1,ADCY3,GNAI1,PRKAR2A,PIK3C2G,TSG101,CDK1,GNAI2,PRKCI,ADCY1,PIK3CB,PPP2R5E,PRKCB
Calcium Signaling	5.05E+00	MYH10,CHRNA1,GRIN2A,MYH6,CHRNA6,TNNT3,GRIN2D,GRIA1,CHRNB1,TNNT2,MYL6B,MYH11,ATP2A2,GRIA4,MYH7B,NFATC1,CABIN1,CAMK2A,CASQ1,RYR3,CREB1,CAMK1G,CASQ2,MYH1,CAMK2B,GRIN2B,GRIN1,TRPC1,TP63,ITPR2,RYR2,HDAC1,SLC8A3,PRKAR2A,ATP2A3,SLC8A2,PPP3CC,TRPC6,ITPR1,MYL7,RCAN1,ATP2B3,ITPR3,CHRNE,MEF2C,GRIA3,CAMK2G
Colorectal Cancer Metastasis Signaling	5.00E+00	MAP2K4,MMP20,JAK1,WNT3,BAD,AXIN1,MMP16,PIK3R1,SOS2,RACK1,GNB5,GNB1,FGFR3,MYC,GNB3,WNT7B,PIK3CG,WNT4,ATM,CASP3,FGFR1,ADRBK2,FGFR2,MMP2,STAT3,TLR9,CDH1,MMP23B,RHOQ,RND3,RHOA,IRS1,FZD6,MAPK10,IL6ST,RELA,MMP14,MMP15,WNT2,GNG7,WNT7A,KL,PIK3C3,DCC,SMAD4,MMP11,CTNNB1,STAT1,PTGER4,WNT9B,ADCY3,PRKAR2A,PIK3C2G,BAX,TCF7L1,WNT10A,ADCY1,MSH6,PIK3CB,PTGS2
GM-CSF Signaling	4.99E+00	PIK3R1,RACK1,SOS2,FGFR3,CAMK2A,KL,PIK3C3,PIK3CG,CISH,HCK,STAT5B,STAT1,ATM,CAMK2B,ETS1,FGFR1,PIK3C2G,FGFR2,STAT3,PPP3CC,TLR9,IRS1,PIK3CB,PRKCB,CAMK2G
Docosahexaenoic Acid (DHA) Signaling	4.96E+00	CASP3,BAD,FGFR1,PIK3R1,PIK3C2G,APAF1,PDPK1,FGFR2,BAX,TLR9,PNPLA2,APP,FGFR3,FOXO1,KL,PIK3CG,IRS1,PIK3C3,PIK3CB,ATM
IL-9 Signaling	4.80E+00	RELA,JAK1,PIK3R1,FGFR1,PIK3C2G,FGFR2,STAT3,TLR9,FGFR3,KL,PIK3C3,IRS1,PIK3CG,CISH,PIK3CB,STAT5B,STAT1,ATM
Role of NFAT in Cardiac Hypertrophy	4.80E+00	MAP2K4,PIK3R1,RACK1,SOS2,GNB5,CSNK1A1,HAND1,CABIN1,FGFR3,GNB1,GNB3,CAMK2A,PIK3CG,ATM,ITPR2,FGFR1,FGFR2,SLC8A2,ITPR1,PPP3CC,TLR9,IRS1,PRKCD,ITPR3,MAPK10,PRKCH,CAMK2G,IL6ST,NOTUM,GNG7,KL,PIK3C3,CAMK1G,PRKCA,CAMK2B,HDAC1,ADCY3,SLC8A3,GNAI1,PRKAR2A,PIK3C2G,GNAI2,RCAN1,PRKCI,ADCY1,PIK3CB,MEF2C,GATA4,PRKCB
Mitotic Roles of Polo-Like Kinase	4.78E+00	ANAPC2,KIF23,FZR1,CDC20,PLK3,PPP2CA,WEE1,PPP2R3B,CDC23,PLK1,ANAPC1,CDK1,SMC1A,SLK,PLK4,HSP90AB1,PLK2,CAPN1,HSP90AA1,PPP2R5E,ANAPC11,KIF11,CDC25A
UVB-Induced MAPK Signaling	4.78E+00	MAP2K4,RPS6KB1,BAD,FGFR1,PIK3R1,PIK3C2G,FGFR2,TLR9,EIF4E,FGFR3,H3F3A/H3F3B,PRKCI,KL,PIK3CG,IRS1,PRKCD,PIK3C3,MAPK10,PIK3CB,PRKCH,ATM,PRKCA,PRKCB

Mitochondrial L-carnitine Shuttle Pathway	4.67E+00	ACSBG1,SLC27A2,CPT1B,ACSL6,ACSL5,CPT2,SLC27A1,SLC27A3,SLC27A4,ACSL1
Aldosterone Signaling in Epithelial Cells	4.46E+00	SGK1,PIK3R1,SOS2,HSPB8,NOTUM,PDPK1,DNAJC10,PIP5K1B,HSPA5,HSPA1L,FGFR3,HSP90AB1,HSPA12B,SLC12A1,KL,PIK3CG,PIK3C3,DNAJB1,AHCY,ATM,PRKCA,ITPR2,SLC12A2,FGFR1,PIK3C2G,FGFR2,DNAJC2,ITPR1,HSPD1,TLR9,HSPA2,HSPA8,PRKCI,PIP5K1C,PRKCD,IRS1,DNAJB11,ITPR3,HSP90AA1,PIK3CB,PRKCH,PIP4K2C,PRKCB
IGF-1 Signaling	4.45E+00	JAK1,BAD,PIK3R1,SOS2,PDPK1,FGFR3,KL,PIK3C3,PIK3CG,IGFBP1,ATM,RPS6KB1,PXN,YWHAB,FGFR1,PIK3C2G,PRKAR2A,FGFR2,IGFBP5,STAT3,TLR9,IGFBP2,GRB10,PRKCI,NOV,FOXO1,IRS1,IGFBP3,PIK3CB,CYR61,SOCS5
Insulin Receptor Signaling	4.42E+00	JAK1,BAD,SGK1,PIK3R1,SOS2,LIPE,PDPK1,PPP1CB,EIF4E,FGFR3,PPP1CC,GYS1,PPP1R10,KL,PIK3CG,INPP5B,PIK3C3,INS,PPP1CA,ATM,RPS6KB1,FGFR1,PIK3C2G,PRKAR2A,FGFR2,VAMP2,ACLY,TLR9,GRB10,GYS2,PRKCI,RHOQ,FOXO1,SYNJ1,INPP5F,IRS1,PIK3CB,INSR
Prolactin Signaling	4.39E+00	PIK3R1,SOS2,PDPK1,FGFR3,MYC,KL,PIK3C3,PIK3CG,PRLR,STAT5B,STAT1,ATM,PRKCA,FGFR1,PIK3C2G,FGFR2,STAT3,TLR9,PRKCI,IRS1,PRKCD,PRKCH,PIK3CB,TCF7,SOCS5,PRKCB
Gluconeogenesis I	4.31E+00	GPI,ALDOB,ENO3,PGAM1,FBP1,PGAM2,ALDOA,GAPDH,ME1,MDH1,GAPDHS,BPGM
Phototransduction Pathway	4.25E+00	RGS9,PDE6G,GUCY2D,GUCA1C,OPN1LW,PRKAR2A,PDE6C,GNB5,CNGA1,CNGA3,GNB1,GNB3,ARR3,RHO,PDC,RGR,GNAT2,CNGB3,OPN4

Table S5 A comprehensive list of genes in proliferation of liver cells, apoptosis of kidney cells and hypertrophy of cardiac muscle

Genes in the proliferation of liver cells	96_slick_Denovo	96h_slick_OnRamp	96h-source	48h-slick	48h-source	24h-slick	24h-source
**IGFBP1** **(insulin like growth factor binding protein 1)**	1.961031	2.042127	0.332148	0.040966	0.059637	0.125486	0.385384
**FGF7** **(fibroblast growth factor 7)**	N/A	2.836242	0.305489	0.768168	0.187485	0.061262	0.007612
**CXCL14** **(C-X-C motif chemokine ligand 14)**	1.108972	1.05091	0.324006	0.475926	0.171579	0.251738	0.187048
**CXCR4** **(C-X-C motif chemokine receptor 4)**	1.637395	1.726862	0.101524	-0.01244	-0.06249	0.014399	0.035989
**PTGS2** **(prostaglandin-endoperoxide synthase 2)**	1.021444	1.018536	0.180669	0.287539	N/A	0.225955	N/A
**EDNRA** **(endothelin receptor type A)**	0.971844	0.866202	-0.066	0.289496	0.118032	-0.04149	0.001957
**HGF** **(hepatocyte growth factor)**	0.624821	0.774913	0.221619	0.252828	0.23401	-0.0236	-0.04022
**CTSB** **(cathepsin B)**	1.041869	0.708098	0.146974	0.004254	0.004713	0.037561	0.097881
**FAH** **(fumarylacetoacetate hydrolase)**	0.462293	0.466168	0.235736	0.559404	0.115301	-0.02867	-0.04282
**SMPD1** **(sphingomyelin phosphodiesterase 1)**	0.957336	0.92369	0.082928	-0.27571	-0.03275	0.100463	-0.02725
**ADIPOQ** **(adiponectin, C1Q and collagen domain containing)**	1.426684	N/A	N/A	N/A	N/A	N/A	N/A
**IKBKB** **(inhibitor of kappa light polypeptide gene enhancer in B-cells, kinase beta)**	0.521847	0.645949	0.136794	-0.06071	-0.02806	0.076825	0.117641
**MMP14** **(matrix metallopeptidase 14)**	0.699211	0.69372	-0.02512	-0.11592	0.001382	0.061371	0.063958
**PPARG** **(peroxisome proliferator activated receptor gamma)**	N/A	1.085901	0.178374	-0.05001	N/A	0.107154	N/A
**C3** **(complement C3)**	N/A	1.088801	0.138384	0.0148	N/A	0.018641	N/A

**NFKBIA** (NFKB inhibitor alpha)	0.574922	0.52987	-0.0492	0.09596	N/A	0.006745	N/A
**F2** (coagulation factor II, thrombin)	0.419508	0.733081	0.395115	-0.49113	N/A	-0.02545	N/A
**STAT1** (signal transducer and activator of transcription 1)	0.453692	0.224154	-0.0445	0.324024	0.098606	-0.02673	-0.02692
**AHR** (aryl hydrocarbon receptor)	N/A	0.520338	0.124935	0.153631	N/A	0.154991	N/A
**PIK3IP1** (phosphoinositide-3-kinase interacting protein 1)	0.385601	0.554003	-0.00774	-0.10401	N/A	0.064349	N/A
**S1PR2** (sphingosine-1-phosphate receptor 2)	0.226437	0.471081	0.055658	0.13762	N/A	-0.02168	N/A
**IL6ST** (interleukin 6 signal transducer)	0.390495	0.36916	-0.00366	-0.01143	0.012819	0.037758	0.033257
**CREB3L3** (cAMP responsive element binding protein 3 like 3)	0.775466	N/A	N/A	N/A	N/A	N/A	N/A
**HBEGF** (heparin binding EGF like growth factor)	0.513463	0.130701	0.013863	0.0377	N/A	0.029593	N/A
**ARNT** (aryl hydrocarbon receptor nuclear translocator)	0.440023	0.236552	-0.0832	0.054041	N/A	0.072184	N/A
**HNF4A** (hepatocyte nuclear factor 4 alpha)	0.27629	0.310617	0.124767	-0.08959	-0.12738	0.100141	0.087169
**XPA** (XPA, DNA damage recognition and repair factor)	N/A	0.353746	0.144023	0.07268	0.045204	0.08226	-0.02761
**FST** (follistatin)	N/A	0.423069	-0.03737	0.009688	0.205528	0.01004	0.036334
**MST1** (macrophage stimulating 1)	N/A	-0.2036	0.193857	0.20598	N/A	0.375325	N/A
**CTNNB1** (catenin beta 1)	0.263111	0.272726	0.031348	0.027992	-0.12738	0.046213	0.016053
**BMP4** (bone morphogenetic protein 4)	0.296213	0.422589	0.128487	-0.2303	N/A	-0.08876	N/A
**STK3** (serine/threonine kinase 3)	0.302148	0.308056	0.060677	0.036782	-0.08194	-0.01642	-0.08116
**CUL3** (cullin 3)	N/A	0.273741	0.218109	0.085529	N/A	-0.07556	N/A
**JUN** (Jun proto-oncogene, AP-1 transcription factor subunit)	0.59726	N/A	N/A	N/A	-0.01639	N/A	-0.08311
**TIMP2** (TIMP metallopeptidase inhibitor 2)	N/A	0.530043	-0.0797	-0.04447	N/A	0.034657	N/A
**ITGAV** (integrin subunit alpha V)	0.140861	0.26532	-0.0517	-0.08104	0.125701	0.016216	0.001219
**LGALS3** (galectin 3)	N/A	0.469208	0.076568	-0.03301	N/A	-0.10402	N/A
**EDNRB** (endothelin receptor type B)	N/A	0.222483	-0.00129	0.074616	-0.12209	0.122762	0.08335
**PPARD** (peroxisome proliferator activated receptor delta)	0.305502	0.071476	0.030432	-0.07811	N/A	0.019021	N/A
**PLG** (plasminogen)	N/A	0.254788	0.166508	-0.15904	N/A	0.01927	N/A
**NGFR** (nerve growth factor receptor)	0.288956	-0.08801	-0.07712	0.005653	0.006333	0.06735	0.077819
**PPARA** (peroxisome proliferator activated receptor alpha)	0.293066	0.044262	-0.06315	0.010038	-0.0274	-0.04401	0.062466

**NCOA5** (nuclear receptor coactivator 5)	N/A	0.28125	0.020959	-0.11413	N/A	0.075827	N/A
**ABCB4** (ATP binding cassette subfamily B member 4)	0.26331	N/A	N/A	N/A	N/A	N/A	N/A
**RARA** (retinoic acid receptor alpha)	N/A	0.297341	0.047655	-0.04989	-0.03711	-0.05235	-0.00391
**SERPINH1** (serpin family H member 1)	-0.47336	-0.00182	-0.09607	0.393148	0.012615	0.331611	0.004832
**PROX1** (prospero homeobox 1)	N/A	-0.27246	-0.11103	0.181824	0.116111	0.081185	0.092278
**SMAD7** (SMAD family member 7)	N/A	0.179956	0.173863	-0.14644	0.038412	-0.10117	-0.09013
**BMP7** (bone morphogenetic protein 7)	N/A	0.183573	-0.08836	0.033179	N/A	-0.11832	N/A
**CTGF** (connective tissue growth factor)	N/A	-0.11387	0.060619	-0.17269	-0.03436	-0.04775	0.272453
**PTN** (pleiotrophin)	0.296811	0.196468	-0.04737	-0.21857	-0.18225	-0.07255	-0.06841
**ITGB1** (integrin subunit beta 1)	0.205025	0.034898	-0.06532	-0.11546	-0.17485	-0.02231	-0.03251
**THBS1** (thrombospondin 1)	N/A	0.088509	-0.06684	-0.2245	0.053828	-0.13262	0.077455
**TOB1** (transducer of ERBB2, 1)	-0.19469	-0.12278	0.145902	0.008906	-0.12483	0.029321	0.002448
**INSR** (insulin receptor)	N/A	-0.11754	-0.07539	-0.03759	-0.01313	0.015627	-0.05285
**YY1AP1** (YY1 associated protein 1)	N/A	N/A	N/A	N/A	-0.16594	N/A	-0.16407
**CDK2** (cyclin dependent kinase 2)	-0.39663	N/A	N/A	N/A	N/A	N/A	N/A
**ODC1** (ornithine decarboxylase 1)	N/A	-0.28155	0.0594	-0.00442	-0.12315	-0.04857	-0.00551
**NCOR1** (nuclear receptor corepressor 1)	-0.32895	-0.20987	-0.07655	0.043521	N/A	0.161354	N/A
**FGFR2** (fibroblast growth factor receptor 2)	-0.48862	-0.13043	0.008444	0.10775	0.168659	-0.0812	-0.02724
**CNR1** (cannabinoid receptor 1)	N/A	-0.21385	-0.13745	-0.12463	-0.08635	0.043886	0.042058
**SPTBN1** (spectrin beta, non-erythrocytic 1)	N/A	-0.44559	-0.02591	-0.04315	N/A	0.004167	N/A
**SKP2** (S-phase kinase associated protein 2)	-0.38115	-0.09559	-0.04566	-0.00748	0.021391	-0.01415	-0.00556
**FOXM1** (forkhead box M1)	-0.53739	N/A	N/A	N/A	N/A	N/A	N/A
**PIK3R1** (phosphoinositide-3-kinase regulatory subunit 1)	-0.57183	0.038126	0.051158	-0.044	-0.09909	0.060207	0.007872
**GSK3B** (glycogen synthase kinase 3 beta)	-0.55379	-0.22204	0.026097	-0.04947	0.0266	0.082392	-0.05348
**MYC** (v-myc avian myelocytomatosis viral oncogene homolog)	-0.30958	-0.16933	-0.15846	0.005962	-0.0666	-0.13777	-0.11575
**SLC7A5** (solute carrier family 7 member 5)	N/A	-0.46174	-0.13969	-0.11819	-0.08632	-0.19379	N/A
**XDH** (xanthine dehydrogenase)	-0.43584	-0.35617	-0.24603	-0.06996	-0.01548	-0.0321	0.010301
**E2F1** (E2F transcription factor 1)	-0.25714	-0.35064	-0.02882	-0.23558	-0.09459	-0.13179	-0.07954
**RPS6KB1** (ribosomal protein S6 kinase B1)	-0.63513	-0.29687	0.020964	-0.1475	0.035777	-0.04839	-0.12001

**COL1A1** (collagen type I alpha 1 chain)	-0.50049	-0.49173	-0.12527	0.071542	0.020896	-0.07278	-0.11117
**TF** (transferrin)	N/A	-0.45256	-0.23819	-0.47748	-0.2189	0.047371	0.029031
**CXCL12** (C-X-C motif chemokine ligand 12)	N/A	-1.11755	0.348455	-0.44533	-0.22833	0.005692	0.032983
**IGFBP1** (insulin like growth factor binding protein 1)	N/A	-0.97674	-0.18461	-0.22446	-0.05661	-0.01013	0.042332

Genes in the apoptosis of kidney cell lines	96_slick_De novo	96h_slick_OnRamp	96h-source	48h-slick	48h-source	24h-slick	24h-source
**BCL6** (B-cell CLL/lymphoma 6)	2.294558	1.682768	0.221556	-0.1745	N/A	0.148275	N/A
**HGF** (hepatocyte growth factor)	0.624821	0.774913	0.221619	0.252828	0.23401	-0.0236	-0.04022
**STAT3** (signal transducer and activator of transcription 3)	0.807008	0.791507	0.106058	0.052942	0.083085	0.016245	-0.02101
**MAP3K5** (mitogen-activated protein kinase kinase kinase 5)	0.551372	0.316298	0.125268	0.187339	-0.00936	0.17903	0.144257
**ECEL1** (endothelin converting enzyme like 1)	N/A	1.10889	0.169817	-0.03562	0.210954	0.010434	-0.03056
**NFKBIA** (NFKB inhibitor alpha)	0.574922	0.52987	-0.0492	0.09596	N/A	0.006745	N/A
**TCF12** (transcription factor 12)	0.248445	0.404569	0.154252	0.221133	0.005833	0.037807	0.001812
**F2** (coagulation factor II, thrombin)	0.419508	0.733081	0.395115	-0.49113	N/A	-0.02545	N/A
**RASSF4** (Ras association domain family member 4)	0.643822	0.270413	0.055089	-0.01501	0.041113	-0.0209	0.027684
**NF2** (neurofibromin 2)	N/A	0.763543	0.163365	0.062604	N/A	-0.0394	N/A
**BAX** (BCL2 associated X, apoptosis regulator)	N/A	0.702169	0.163665	0.003094	N/A	0.026228	N/A
**SREBF2** (sterol regulatory element binding transcription factor 2)	N/A	0.497711	0.058523	0.090798	0.032348	0.118033	-0.01151
**CAT** (catalase)	N/A	0.605675	0.121069	0.105784	N/A	-0.05376	N/A
**CASP3** (caspase 3)	0.437424	0.393741	-0.08941	-0.01898	N/A	0.044251	N/A
**ATF6** (activating transcription factor 6)	0.25984	0.282648	0.042235	-0.05233	0.027754	0.034087	0.123021
**FOXO1** (forkhead box O1)	N/A	0.691292	0.051287	-0.05764	-0.03876	-0.01499	0.084112
**VTN** (vitronectin)	N/A	0.36116	0.121318	0.06024	0.104731	0.041863	0.023971
**PIK3CA** (phosphatidylinositol-4,5-bisphosphate 3-kinase catalytic subunit alpha)	N/A	-0.01688	0.025215	0.276501	0.288781	-0.00805	0.100492
**ABCC1** (ATP binding cassette subfamily C member 1)	0.406475	0.147779	-0.0282	0.166461	0.030576	-0.04075	-0.02012
**HSPA5** (heat shock protein family A (Hsp70) member 5)	N/A	0.194962	0.198438	0.166541	0.028165	0.072414	-0.00284
**STK26** (serine/threonine protein kinase 26)	N/A	0.469386	0.00553	0.006319	0.034678	0.038814	0.084892
**CHMP5** (charged	0.592402	N/A	N/A	N/A	N/A	N/A	N/A

**multivesicular body protein 5)**							
**DDX17 (DEAD-box helicase 17)**	0.42952	0.045468	0.055024	0.018214	0.015275	0.004762	0.019728
**RNF13 (ring finger protein 13)**	0.245021	0.25124	-0.04917	0.068386	0.091265	-0.01357	-0.0444
**UNC5C (unc-5 netrin receptor C)**	N/A	0.061704	0.096947	0.162495	0.224221	-0.08336	0.072368
**CTNNB1 (catenin beta 1)**	0.263111	0.272726	0.031348	0.027992	-0.12738	0.046213	0.016053
**TNFRSF19 (TNF receptor superfamily member 19)**	0.188508	0.269331	0.00146	-0.02451	0.047195	-0.0635	0.101689
**IL17RD (interleukin 17 receptor D)**	0.478509	-0.07696	-0.01532	0.049577	0.020694	0.029924	-0.00779
**RHOG (ras homolog family member G)**	0.209271	0.340107	0.008911	-0.12584	N/A	0.040696	N/A
**PSEN2 (presenilin 2)**	N/A	0.396866	0.067997	-0.04658	-0.12539	0.057979	0.114311
**LRP5 (LDL receptor related protein 5)**	N/A	0.111777	0.052865	-0.07915	0.141013	0.090163	0.128424
**IGFBP3 (insulin like growth factor binding protein 3)**	N/A	0.426795	-0.0007	-0.02387	-0.0614	-0.01837	0.098734
**ITGAV (integrin subunit alpha V)**	0.140861	0.26532	-0.0517	-0.08104	0.125701	0.016216	0.001219
**MAP1S (microtubule associated protein 1S)**	N/A	0.267085	0.126942	0.002074	N/A	0.000122	N/A
**BAD (BCL2 associated agonist of cell death)**	N/A	0.374987	-0.03295	0.074832	N/A	-0.02608	N/A
**PPARD (peroxisome proliferator activated receptor delta)**	0.305502	0.071476	0.030432	-0.07811	N/A	0.019021	N/A
**TJP2 (tight junction protein 2)**	0.311715	0.049263	-0.04205	0.020558	-0.066	0.037677	0.035788
**ZMYND11 (zinc finger MYND-type containing 11)**	N/A	-0.09956	-0.04842	0.151087	0.164782	0.054063	0.12105
**RIPK1 (receptor interacting serine/threonine kinase 1)**	0.26447	0.078508	-0.00766	-0.01128	0.004537	-0.01384	-0.01812
**ZNF512B (zinc finger protein 512B)**	0.372474	-0.18363	-0.06662	0.115796	N/A	-0.03889	N/A
**TFRC (transferrin receptor)**	N/A	-0.07313	0.0198	0.100538	0.107968	-0.03595	0.032722
**EZR (ezrin)**	-0.27029	0.236371	0.062846	0.050913	-0.00555	-0.02651	0.073939
**FGFR4 (fibroblast growth factor receptor 4)**	0.212335	-0.05061	0.037404	-0.12988	N/A	0.006777	N/A
**SMAD7 (SMAD family member 7)**	N/A	0.179956	0.173863	-0.14644	0.038412	-0.10117	-0.09013
**RBP4 (retinol binding protein 4)**	N/A	0.51165	0.164203	-0.48076	N/A	-0.14353	N/A
**GRIN2B (glutamate ionotropic receptor NMDA type subunit 2B)**	N/A	-0.22009	0.005378	0.161232	0.176302	-0.01203	-0.07506
**ITCH (itchy E3 ubiquitin protein ligase)**	N/A	-0.16933	-0.06255	-0.03882	-0.06859	0.12823	0.23229
**ILK (integrin linked kinase)**	N/A	0.12497	-0.08322	-0.00554	N/A	-0.0271	N/A
**PAK1 (p21 (RAC1) activated kinase 1)**	-0.3029	0.19265	-0.00108	0.052288	0.07384	0.017184	-0.02556
**PKD1 (polycystin 1, transient receptor potential channel interacting)**	0.542496	-0.3547	-0.18674	-0.02635	0.045332	-0.08138	-0.09312

**ELOVL4** (ELOVL fatty acid elongase 4)	-0.31604	-0.05041	0.019684	0.069469	0.022078	-0.01135	0.105719
**ITGB1** (integrin subunit beta 1)	0.205025	0.034898	-0.06532	-0.11546	-0.17485	-0.02231	-0.03251
**SLK** (STE20 like kinase)	-0.25614	-0.18751	0.025097	0.05513	0.149628	-0.01954	-0.10988
**UNC5A** (unc-5 netrin receptor A)	N/A	-0.40025	-0.07986	0.079073	0.10061	-0.026	-0.04469
**PKN2** (protein kinase N2)	N/A	-0.23533	-0.06008	-0.09494	-0.02567	0.034376	0.001755
**CUL4B** (cullin 4B)	N/A	-0.20992	0.006079	-0.01718	-0.07171	-0.03094	-0.07592
**GLS** (glutaminase)	-0.431	N/A	N/A	N/A	N/A	N/A	N/A
**AATF** (apoptosis antagonizing transcription factor)	-0.67618	N/A	N/A	N/A	N/A	N/A	N/A
**DAP3** (death associated protein 3)	-0.30399	-0.32074	-0.02623	-0.09243	N/A	0.022442	N/A
**GRB10** (growth factor receptor bound protein 10)	-0.63103	-0.39386	-0.12549	0.204545	0.191925	-0.01921	0.040813
**CDK1** (cyclin dependent kinase 1)	N/A	-0.32743	0.013911	-0.1163	-0.16591	-0.05304	-0.11115
**GSN** (gelsolin)	-0.78665	0.134984	-0.01264	-0.07841	-0.14232	-0.00971	-0.03673
**GSK3B** (glycogen synthase kinase 3 beta)	-0.30958	-0.16933	-0.15846	0.005962	-0.0666	-0.13777	-0.11575
**STRA6** (stimulated by retinoic acid 6)	N/A	-1.04227	-0.01631	0.056293	-0.00452	0.003264	0.047898
**PRMT1** (protein arginine methyltransferase 1)	-0.39847	-0.36868	-0.07279	0.03021	N/A	-0.14869	N/A
**MYC** (v-myc avian myelocytomatosis viral oncogene homolog)	N/A	-0.46174	-0.13969	-0.11819	-0.08632	-0.19379	N/A
**PSIP1** (PC4 and SFRS1 interacting protein 1)	-0.44666	-0.29424	-0.15178	-0.14511	0.042048	0.000312	-0.03572
**IRS1** (insulin receptor substrate 1)	-0.62713	-0.74706	-0.28824	-0.24097	0.041842	-0.01858	0.131675
**CA4** (carbonic anhydrase 4)	-0.89532	-0.7675	-0.15022	-0.09293	N/A	-0.00486	N/A

Genes in the hypertrophy of cardiac muscle	96_slick_Denovo	96h_slick_OnRamp	96h-source	48h-slick	48h-source	24h-slick	24h-source
**STAT3** (signal transducer and activator of transcription 3)	0.971844	0.866202	-0.066	0.289496	0.118032	-0.04149	0.001957
**MAP3K5** (mitogen-activated protein kinase kinase kinase 5)	0.624821	0.774913	0.221619	0.252828	0.23401	-0.0236	-0.04022
**ADIPOQ** (adiponectin, C1Q and collagen domain containing)	0.807008	0.791507	0.106058	0.052942	0.083085	0.016245	-0.02101
**IKBKB** (inhibitor of kappa light polypeptide gene enhancer in B-cells, kinase beta)	0.551372	0.316298	0.125268	0.187339	-0.00936	0.17903	0.144257
**NFKBIA** (NFKB inhibitor alpha)	1.426684	N/A	N/A	N/A	N/A	N/A	N/A
**ECE1** (endothelin converting enzyme 1)	0.521847	0.645949	0.136794	-0.06071	-0.02806	0.076825	0.117641
**IL6ST** (interleukin 6 signal transducer)	0.574922	0.52987	-0.0492	0.09596	N/A	0.006745	N/A
**HBEGF** (heparin binding	0.192593	0.296006	0.070892	0.15566	N/A	0.14992	N/A

EGF like growth factor)				3		3	
**BMP4** (bone morphogenetic protein 4)	0.390495	0.36916	-0.00366	-0.01143	0.012819	0.037758	0.033257
**STK3** (serine/threonine kinase 3)	0.513463	0.130701	0.013863	0.0377	N/A	0.029593	N/A
**IGF2R** (insulin like growth factor 2 receptor)	0.296213	0.422589	0.128487	-0.2303	N/A	-0.08876	N/A
**FSTL3** (follistatin like 3)	0.302148	0.308056	0.060677	0.036782	-0.08194	-0.01642	-0.08116
**PRKCB** (protein kinase C beta)	0.476799	0.451805	0.023947	-0.25316	-0.14501	0.023156	-0.0721
**DUSP1** (dual specificity phosphatase 1)	N/A	1.029101	-0.06689	-0.4654	N/A	-0.01207	N/A
**CABIN1** (calcineurin binding protein 1)	N/A	0.171513	-0.03771	-0.17017	-0.01801	0.1989	0.284938
**MYOD1** (myogenic differentiation 1)	0.243023	0.180264	0.094669	-0.21967	N/A	0.07186	N/A
**AKAP1** (A-kinase anchoring protein 1)	-0.21107	-0.2711	0.015984	0.00404	0.114633	0.120401	0.081749
**MEF2C** (myocyte enhancer factor 2C)	0.534087	-0.43205	-0.00725	-0.16206	-0.10406	-0.05443	0.018249
**PARP1** (poly(ADP-ribose) polymerase 1)	-0.22848	-0.07043	-0.04834	0.023203	-0.01614	0.023051	-0.12955
**MYBPC3** (myosin binding protein C, cardiac)	-0.20957	-0.33001	-0.18218	0.066919	N/A	-0.04846	N/A
**PRMT5** (protein arginine methyltransferase 5)	N/A	-0.30168	0.012349	0.034896	-0.14089	-0.17221	-0.15675
**GSK3B** (glycogen synthase kinase 3 beta)	-0.43305	-0.24377	-0.04632	-0.01157	-0.05304	0.010457	-0.01812
**FHL2** (four and a half LIM domains 2)	N/A	-0.5772	-0.13604	0.01939	-0.09413	-0.01632	-0.09266
**TTN** (titin)	-0.30958	-0.16933	-0.15846	0.005962	-0.0666	-0.13777	-0.11575
**STAT3** (signal transducer and activator of transcription 3)	-0.21454	-0.23281	-0.05638	-0.42656	-0.15781	0.082816	0.042815
**MAP3K5** (mitogen-activated protein kinase kinase kinase 5)	-1.12527	N/A	N/A	N/A	N/A	N/A	N/A

Table S6 A list of toxicity pathways and DEGs responsible for their enrichment by IPA-Tox

Ingenuity Toxicity Lists	-log(p-value)	Downregulated	Upregulated	Overlap with dataset	Molecules
**Liver Proliferation**	7.74E00	15/228 (7%)	37/228 (16%)	176/228 (77%)	PPARA,BMP4,XDH,PIK3R1,ODC1,F2,ARNT,IKBKB,CXCL14,ITGAV,SLC7A5,GSK3B,IGFBP1,HNF4A,RPS6KB1,HBEGF,SKP2,SCP2,E2F1,CTSB,MDK,IL6ST,SPTBN1,MMP14,PTN,ADIPOQ,SAT1,SMPD1,YY1AP1,JUN,NFKBIA,NGFR,HGF,RB1CC1,NCOR1,CTNNB1,STAT1,PIK3IP1,ITGB1,S1PR2,PPARD,CXCR4,CREB3L3,TOB1,STK3,FOXM1,FAH,CBS/CBSL,ABCB4,SERPINH1,EDNRA,PTGS2
**Cholesterol Biosynthesis**	7.5E00	1/16 (6%)	10/16 (63%)	5/16 (31%)	FDPS,FDFT1,EBP,DHCR7,PMVK,ACAT2,ACAT1,LSS,HMGCR,HMGCS1,SC5D
**Cardiac Hypertrophy**	6.02E00	32/435 (7%)	45/435 (10%)	358/435 (82%)	PPARA,APOB,BMP4,TCF15,RAB2A,PIK3R1,TNNT2,PBX1,CORIN,TPM1,ATP2A2,ABCA1,CABIN1,IKBKB,FHL2,FKBP1B,LCAT,PIK3CG,GSK3B,CA

					SQ2,HBEGF,MMP2,MYH7,STAT3,GSN,TTN,PFKM,ANKRD1,TRIM63,DUSP1,PRKCD,CTSB,HPRT1,IL6ST,NOS1,MYH6,ADAM17,PDGFA,ADIPOQ,MAP3K5,KCNQ1,KCNJ11,EIF4EBP1,NFKBIA,JUN,SP3,PARK7,PPP3CB,HGF,PRKCE,ECE1,CTNNB1,ACE,RASSF1,VAV2,EPAS1,SLC25A4,TRIM55,PPARD,MYOD1,XIRP1,STK3,IGF2R,PNPLA2,SEMA3A,NF1,NT5E,LAMA4,KLF5,EDNRA,MEF2C,PTGS2,CTSC,HMGCR,COL9A2,MYBPC3,AKAP1
**Liver Necrosis/Cell Death**	4.79E00	18/289 (6%)	35/289 (12%)	236/289 (82%)	PPARA,PIK3R1,ARNT,OCLN,IKBKB,TNIP1,ITGAV,GSK3B,IGFBP1,HNF4A,CASP3,HBEGF,STAT3,IRF5,ADAR,E2F1,CTSB,IL6ST,MME,SIGIRR,SPTBN1,ATF7,ADIPOQ,UGCG,SMPD1,USP2,ATP2A1,ALDH2,YY1AP1,NFKBIA,JUN,FLNA,NGFR,HGF,RB1CC1,STAT1,SLC25A4,MYD88,PLK1,SOD1,STK3,SH3BP5,FAH,NFE2L1,BUB1,LDLR,NF1,ABCB4,CBS/CBSL,SERPINH1,PTGS2,DNM1L,A2M
**Renal Necrosis/Cell Death**	4.67E00	29/519 (6%)	54/519 (10%)	436/519 (84%)	CCNI,SUV39H1,CA4,ABCC10,BCL6,APRT,F2,NTN1,STMN1,FDFT1,RNF13,PAK1,RHOG,EZR,ABCC1,FGFR4,ITGAV,GSK3B,HK1,ATG7,TJP2,CASP3,DCN,HBEGF,STAT3,GSN,PFKM,DCTN2,PKD1,DUSP1,IRS1,PRKCD,RASSF4,NMNAT3,GAPDH,NEIL1,CHMP5,SFRP1,NOS1,AATF,IL17RD,ERN1,ATF6,DDX17,MAP3K5,BECN1,HNRNPK,SIRT4,TCF12,ALKBH8,HK2,NFKBIA,ANXA5,NOP58,HGF,VCP,DAP3,PRKCE,SLC40A1,ZNF512B,CTNNB1,ATP13A2,RASSF1,DNASE1,ITGB1,HNRNPU,MYD88,PPARD,GLS,ELOVL4,MAN2A1,HIP1,SOD1,PSIP1,PRMT1,GRB10,SLK,SLC7A9,RIPK1,PTGS2,ABCC3,TNFRSF19,PTGR1
**Increases Liver Hyperplasia/Hyperproliferation**	3.65E00	13/103 (13%)	10/103 (10%)	80/103 (78%)	PPARA,SPTBN1,MYD88,CXCR4,HMMR,NCAPG,FAM120A,STAT3,IQGAP1,FOXM1,MEN1,PSME3,VCAN,ODC1,SKP2,BUB1,IKBKB,JUN,HGF,ACSS2,E2F1,HMGCR,CTNNB1
**Hypoxia-Inducible Factor Signaling**	3.29E00	8/70 (11%)	9/70 (13%)	53/70 (76%)	PXN,UBE2A,COPS5,PIK3R1,CSNK1D,EIF2S1,EIF2S2,ELAVL1,ARNT,IKBKB,JUN,HSP90AB1,HIF1AN,HSP90AA1,KDR,UBE2E1,EIF1AX
**Cardiac Necrosis/Cell Death**	3.1E00	19/273 (7%)	26/273 (10%)	228/273 (84%)	IL6ST,MYH6,XDH,PTN,ADIPOQ,MAP3K5,E2F3,BECN1,IQGAP1,NTN1,FSTL1,IKBKB,HK2,PARK7,PHB,PPP1R10,HGF,MANF,HSPE1,GSK3B,CTNNB1,MAPKAPK2,STAT1,RASSF1,AIFM1,HK1,CALR,CASP3,UBE4B,CXCR4,TXNIP,CSK,SERPINF1,MMP2,STAT3,GSN,SH3BP5,PNPLA2,NUB1,PRKCD,E2F1,GAPDH,MDK,NECTIN2,MYBPC3
**Cardiac Fibrosis**	2.98E00	12/193 (6%)	22/193 (11%)	159/193 (82%)	NOS1,PPARA,MYH6,TNNT2,RRM2B,CTNNA1,PPP1CB,MAP3K5,BCL6,BECN1,ATP2A2,LPL,GSK3B,DAG1,ACE,RASSF1,VAV2,ITGB1,PTX3,ETS1,ENTPD1,XIRP1,HBEGF,STAT3,STK3,PNPLA2,NF1,LAMA4,RERE,KLF5,PTGS2,ACSL1,MYBPC3,NECTIN2
**Genes Upregulated in Response to Proteinuria-induced Oxidative Stress in Renal Proximal Tubule Cells (Human)**	2.75E00	1/10 (10%)	4/10 (40%)	5/10 (50%)	HSP90AA1,MAPK12,POLD1,SOD3,GSTT1
**Mitochondrial Dysfunction**	2.48E00	8/176 (5%)	22/176 (13%)	146/176 (83%)	PRDX5,XDH,CPT1B,ACO2,RHOT2,TRAK1,MT-CO2,NDUFA1,NDUFB9,NDUFS1,PARK7,MT-CO3,OGDH,MT-ND2,AIFM1,SDHA,NDUFV1,UCP2,CASP3,ATP5A1,MT-ND4,NDUFAF2,MAPK12,FIS1,MT-ND5,MT-ND1,NDUFV2,APH1B,BACE2,COX15
**TR/RXR Activation**	2.02E00	8/98 (8%)	10/98 (10%)	80/98 (82%)	PIK3C2B,UCP2,PIK3R1,NCOA6,NCOA4,ATP2A1,KLF9,F10,LDLR,SREBF1,PIK3CG,FGFR4,IRS1,FASN,PIK3C3,ACACA,NCOR1,STRBP
**PPARα/RXRα Activation**	2E00	15/182 (8%)	14/182 (8%)	153/182 (84%)	PPARA,PRKAB1,PRKAB2,CPT1B,TGFBR3,ADIPOQ,GNA14,AP2A2,ABCA1,PRKAG1,PLCH1,IKBKB,NFKBIA,JUN,HSP90AB1,FASN,LPL,NCOR1,STAT5B,ACOX1,NCOA6,IL1R1,CAND1,GK,IRS1,HSP90AA1,SLC27A1,MEF2C,MAP4K4
**LXR/RXR Activation**	1.91E00	9/123 (7%)	12/123 (10%)	102/123 (83%)	HPX,APOB,APOH,AHSG,SERPINF1,IL1R1,ABCA1,SERPINF2,FDFT1,LDLR,LCAT,SREBF1,NGFR,FASN,LPL,ACACA,NCOR1,PLTP,PTGS2,HMGCR,CYP51A1
**Hepatic Fibrosis**	1.89E00	6/101 (6%)	12/101 (12%)	83/101 (82%)	LTBP2,COL4A1,PDGFA,DCN,MMP14,MMP13,IGFBP5,MMP2,COL1A2,COL5A1,TNC,SREBF1,HGF,ECE1,LTBP4,IGFBP1,A2M,Hspg2
**Mechanism of Gene Regulation by Peroxisome Proliferators via PPARα**	1.83E00	4/95 (4%)	13/95 (14%)	78/95 (82%)	PPARA,PPARD,PDGFA,CPT1B,PIK3R1,ACOX1,IL1R1,IKBKB,NFKBIA,JUN,HSP90AB1,DUSP1,LPL,HSP90AA1,NCOR1,PTGS2,STAT5B
**NRF2-mediated Oxidative Stress Response**	1.81E00	8/245 (3%)	28/245 (11%)	209/245 (85%)	PRDX1,PIK3R1,MAP3K5,SOD3,GSTT1,JUN,HSP90AB1,PIK3C3,PIK3CG,ABCC1,FGFR4,VCP,UBE2K,PRKCE,DNAJA3,GSK3B,DNAJB1,CBR1,ACTA1,PIK3C2B,CYP1A1,DNAJB12,SOD1,DNAJB14,TXNRD1,GSTO1,DNAJC11,PRKCI,PRKCD,IRS1,CCT7,HSP90AA1,CYP4A11,ENC1,DNAJC7,MGST3
**FXR/RXR Activation**	1.8E00	10/126 (8%)	11/126 (9%)	105/126 (83%)	PPARA,HPX,APOB,PKLR,APOH,FETUB,AHSG,SERPINF1,SLC22A7,MAPK12,SERPINF2,CYP8B1,ABCB4,LCAT,SREBF1,FGFR4,FASN,LPL,FBP1,PLTP,HNF4A
**Increases Liver Steatosis**	1.69E00	4/91 (4%)	12/91 (13%)	75/91 (82%)	PRKAB1,ATP5A1,RGS16,ADIPOQ,MAP3K5,TRPV1,PSME3,ARNT,IKBKB,SOAT2,ACOT11,DUSP1,SREBF1,PLIN2,HIF1AN,RPTOR
**VDR/RXR Activation**	1.61E00	1/78 (1%)	13/78 (17%)	64/78 (82%)	SERPINB1,CSNK2A1,PPARD,PDGFA,IGFBP5,PSMC5,YY1,PRKCI,PRKCD,MXD1,PRKCE,NCOR1,IGFBP1,SEMA3B
**RAR Activation**	1.54E00	6/190 (3%)	22/190 (12%)	162/190 (85%)	CSNK2A1,PIK3R1,Rdh7,MAP3K5,RBP1,PRKAG1,PSMC5,TNIP1,LRAT,JUN,PIK3CG,RDH14,PRKCE,NCOR1,NR2F6,STAT5B,MAPKAPK2,SMAD1,RDH13,DHRS3,CSK,MAPK12,SMARCD3,PRMT1,PRKCI,DUSP1,PRKC

					D,RDH12
**Long-term Renal Injury Anti-oxidative Response Panel (Rat)**	1.52E00	2/18 (11%)	3/18 (17%)	13/18 (72%)	CBS/CBSL,BLVRA,CP,SOD1,SOD3
**p53 Signaling**	1.47E00	8/112 (7%)	10/112 (9%)	94/112 (84%)	PIK3C2B,TP63,TOPBP1,PIK3R1,RRM2B,CSNK1D,SERPINE2,JUN,PIK3C3,PIK3CG,FASN,IRS1,FGFR4,PPP1R13B,E2F1,GSK3B,TRIM29,CTNNB1
**Long-term Renal Injury Pro-oxidative Response Panel (Rat)**	1.44E00	2/13 (15%)	2/13 (15%)	9/13 (69%)	XDH,HIF3A,HPRT1,FMO5
**Hepatic Cholestasis**	1.41E00	2/162 (1%)	22/162 (14%)	138/162 (85%)	PPARA,TJP2,MYD88,SLC22A7,IL1R1,MAPK12,PRKAG1,CYP8B1,ATP8B1,SLCO1C1,IKBKB,PRKCI,JUN,NFKBIA,ABCB4,SLCO3A1,SREBF1,NGFR,FGFR4,ABCC1,PRKCD,PRKCE,HNF4A,ABCC3
**Acute Renal Failure Panel (Rat)**	1.33E00	5/62 (8%)	6/62 (10%)	51/62 (82%)	MYO5A,GAS2,JUN,ANXA1,TAGLN,ANXA2,IGFBP5,IGFBP1,COL18A1,A2M,EIF1AX
**Genes associated with Chronic Allograft Nephropathy (Human)**	1.26E00	1/21 (5%)	4/21 (19%)	16/21 (76%)	TNC,COL4A1,PDGFA,MMP2,ITGB6
**Nongenotoxic Hepatocarcinogenicity Biomarker Panel**	1.18E00	4/22 (18%)	1/22 (5%)	17/22 (77%)	CITED4,LOC102724788/PRODH,TRNT1,ANXA2,USP2
**LPS/IL-1 Mediated Inhibition of RXR Function**	1.09E00	13/253 (5%)	20/253 (8%)	220/253 (87%)	PPARA,ALDH4A1,CPT1B,FMO5,SOD3,ABCA1,GSTT1,ALDH1L1,JUN,NGFR,ACSL5,CHST3,FABP7,SULT1C3,HMGCS1,ALDH6A1,ALDH7A1,MYD88,ACOX1,IL1R1,GSTO1,HS3ST3B1,FABP2,ALDH1L2,SREBF1,SMOX,SLC27A1,PLTP,ALDH16A1,ABCC3,CYP4A11,ACSL1,MGST3
**Aryl Hydrocarbon Receptor Signaling**	1.07E00	11/159 (7%)	11/159 (7%)	137/159 (86%)	TRIP11,ALDH4A1,CYP1A1,CCNE2,RBL1,GSTT1,GSTO1,ARNT,CCNA2,ALDH1L1,JUN,ALDH1L2,HSP90AB1,FASN,E2F1,HSP90AA1,DHFR,ALDH16A1,ALDH6A1,MGST3,ALDH7A1,MCM7
**Increases Cardiac Dysfunction**	1.02E00	3/39 (8%)	4/39 (10%)	32/39 (82%)	PPARA,PTX3,ANGPTL4,PIK3CG,E2F1,SLC27A1,MAP3K5
**Increases Renal Proliferation**	9.49E-01	7/139 (5%)	12/139 (9%)	120/139 (86%)	ITGB1,DLGAP5,CDCP1,MMP14,TTLL4,STAT3,NTN1,SKP2,CSNK1E,JUN,HGF,NOP58,E2F1,BICC1,KLF5,CHKA,LARP1,PTGS2,CTNNB1
**Increases Glomerular Injury**	9.36E-01	3/89 (3%)	10/89 (11%)	76/89 (85%)	SLK,ITGB1,BMP4,CASP3,PARK7,SREBF1,MYD88,TXNIP,PRKCD,PTGS2,CTNNB1,DDR1,SKP2
**Oxidative Stress**	9.18E-01	1/57 (2%)	8/57 (14%)	48/57 (84%)	JUN,PRDX5,DUSP1,PRDX1,XDH,STAT3,SOD1,NFE2L1,SOD3
**Cell Cycle: G1/S Checkpoint Regulation**	8.87E-01	10/66 (15%)	0/66 (0%)	56/66 (85%)	CCNE2,MAX,PA2G4,SUV39H1,E2F1,GSK3B,RBL1,E2F3,SKP2,CDC25A
**Decreases Depolarization of Mitochondria and Mitochondrial Membrane**	8.75E-01	3/27 (11%)	2/27 (7%)	22/27 (81%)	PAK1,VPS13A,ABCC1,DNM1L,ATP2A2
**Renal Proximal Tubule Toxicity Biomarker Panel (Rat)**	8.75E-01	2/27 (7%)	3/27 (11%)	22/27 (81%)	HSP90AA1,IGFBP1,SLC38A3,SLC15A2,OCLN
**Xenobiotic Metabolism Signaling**	8.08E-01	15/352 (4%)	27/352 (8%)	310/352 (88%)	ALDH4A1,LTA4H,PIK3R1,EPHX2,PPP2R3B,MAP3K5,FMO5,SOD3,GSTT1,ARNT,ALDH1L1,CAMK2A,HSP90AB1,FGFR4,PIK3CG,PIK3C3,CHST3,PRKCE,SULT1C3,CYP51A1,ALDH6A1,ALDH7A1,CAMK2B,PIK3C2B,CYP1A1,UGT8,MAPK12,GSTO1,HS3ST3B1,PRKCI,ALDH1L2,PPP2R3A,SMOX,PRKCD,IRS1,HSP90AA1,PPP2R5E,ALDH16A1,ABCC3,DNAJC7,MGST3,CAMK2G
**Recovery from Ischemic Acute Renal Failure (Rat)**	7.85E-01	1/14 (7%)	2/14 (14%)	11/14 (79%)	COL4A1,SGK1,RBP1
**Positive Acute Phase Response Proteins**	7.35E-01	2/30 (7%)	3/30 (10%)	25/30 (83%)	HPX,CP,A2M,SERPINF2,HMOX2
**Swelling of Mitochondria**	7.2E-01	1/15 (7%)	2/15 (13%)	12/15 (80%)	PRKCE,SOD1,AIFM1
**Hormone Receptor Regulated Cholesterol Metabolism**	7.17E-01	1/8 (13%)	1/8 (13%)	6/8 (75%)	LDLR,ABCA1
**Negative Acute Phase Response Proteins**	7.17E-01	1/8 (13%)	1/8 (13%)	6/8 (75%)	ITIH2,AHSG
**Vasopressin-induced Genes in Inner Medullary Renal Collecting Duct Cells (Rat)**	7.17E-01	1/8 (13%)	1/8 (13%)	6/8 (75%)	SGK1,WNK1

**Fatty Acid Metabolism**	6.81E-01	8/117 (7%)	7/117 (6%)	102/117 (87%)	ALDH4A1,CYP1A1,ACAT2,CPT1B,ACOX1,ACADSB,ALDH2,AUH,ACSL5,ACAT1,SLC27A1,CYP4A11,ACSL1,CYP51A1,ALDH7A1
**Increases Transmembrane Potential of Mitochondria and Mitochondrial Membrane**	6.15E-01	3/50 (6%)	4/50 (8%)	43/50 (86%)	HSPA4,CASP3,PHB,HGF,PRKCE,DNAJB1,GSN
**Genes Downregulated in Response to Chronic Renal Failure (Rat)**	5.68E-01	0/10 (0%)	2/10 (20%)	8/10 (80%)	CYP1A1,SLC22A7
**Renal Ischemic Resistance Panel (Rat)**	5.68E-01	2/10 (20%)	0/10 (0%)	8/10 (80%)	CCNF,FOXM1
**Increases Renal Damage**	5.12E-01	1/81 (1%)	9/81 (11%)	71/81 (88%)	ITGB1,BMP4,SREBF1,MYD88,TXNIP,MDK,IL1R1,ITGB6,CFP,CTNNB1
**Primary Glomerulonephritis Biomarker Panel (Human)**	5.09E-01	1/11 (9%)	1/11 (9%)	9/11 (82%)	TYMS,HBEGF
**Biogenesis of Mitochondria**	4.78E-01	1/20 (5%)	2/20 (10%)	17/20 (85%)	EPAS1,DNAJA3,MAN2A1
**Glutathione Depletion - Phase II Reactions**	4.78E-01	0/20 (0%)	3/20 (15%)	17/20 (85%)	GSTO1,GSTT1,MGST3
**Increases Cardiac Proliferation**	4.64E-01	1/47 (2%)	5/47 (11%)	41/47 (87%)	CASZ1,ADIPOQ,HBEGF,STAT3,STAT1,CTNNB1
**Glutathione Depletion - CYP Induction and Reactive Metabolites**	4.57E-01	0/12 (0%)	2/12 (17%)	10/12 (83%)	ABCC3,GSTT1
**Persistent Renal Ischemia-Reperfusion Injury (Mouse)**	4.38E-01	1/30 (3%)	3/30 (10%)	26/30 (87%)	LTBP2,CTSS,MMP14,SOX9
**Increases Liver Damage**	3.85E-01	1/117 (1%)	12/117 (10%)	104/117 (89%)	IL6ST,PPARA,MYD88,SMPD1,STAT3,IL1R1,SH3BP5,F2,IKBKB,CTSB,PTGS2,DNM1L,STAT1
**Genes Upregulated in Response to Chronic Renal Failure (Rat)**	3.82E-01	0/5 (0%)	1/5 (20%)	4/5 (80%)	ABCC3
**Increases Heart Failure**	3.78E-01	1/23 (4%)	2/23 (9%)	20/23 (87%)	TRIM63,PIK3CG,PAX3
**Cytochrome P450 Panel - Substrate is a Sterol (Human)**	3.72E-01	1/14 (7%)	1/14 (7%)	12/14 (86%)	CYP51A1,CYP8B1
**Cytochrome P450 Panel - Substrate is a Sterol (Mouse)**	3.72E-01	1/14 (7%)	1/14 (7%)	12/14 (86%)	CYP51A1,CYP8B1
**Cytochrome P450 Panel - Substrate is a Sterol (Rat)**	3.72E-01	1/14 (7%)	1/14 (7%)	12/14 (86%)	CYP51A1,CYP8B1
**Renal Inorganic Phosphate Homeostasis (Mouse)**	3.24E-01	1/6 (17%)	0/6 (0%)	5/6 (83%)	ATP1A2
**Renal Glomerulus Panel (Human)**	2.77E-01	1/17 (6%)	1/17 (6%)	15/17 (88%)	PTPRO,DAG1
**Decreases Permeability Transition of Mitochondria and Mitochondrial Membrane**	2.77E-01	1/7 (14%)	0/7 (0%)	6/7 (86%)	OGDH
**Glutathione Depletion - Hepatocellular Hypertrophy**	2.39E-01	0/8 (0%)	1/8 (13%)	7/8 (88%)	ABCC3
